# Neuroimaging Correlates of the NIH Toolbox Cognition and Trail Making Tests: Normative Benchmarks in Healthy Aging

**DOI:** 10.3390/ctn10010005

**Published:** 2026-02-03

**Authors:** Cuiping Yuan, Hector Acosta-Rodriguez, Nahla M. H. Elsaid, Clara F. Weber, Pratheek Bobba, Anh T. Tran, Ajay Malhotra, Seyedmehdi Payabvash

**Affiliations:** 1 Department of Radiology, Affiliated Hospital of Nanjing University of Chinese Medicine, Jiangsu Province Hospital of Chinese Medicine, Nanjing 210029, China;; 2 Department of Radiology and Biomedical Imaging, Yale School of Medicine, New Haven, CT 06520, USA; 3 Developing Brain Institute, Children’s National, Washington, DC 20010, USA; 4 Social Neuroscience Laboratory, Department of Psychiatry and Psychotherapy, University of Lübeck, 23562 Lübeck, Germany; 5 Department of Radiology, NewYork-Presbyterian Hospital at Columbia University Irving Medical Center, Columbia University, New York, NY 10032, USA

**Keywords:** human connectome project, NIH toolbox, NODDI, fluid cognition, crystallized cognition, trail making test

## Abstract

The National Institutes of Health (NIH) Toolbox cognition battery and Trail Making Tests (TMT) are widely used to quantify cognitive aging and to detect early cognitive vulnerability in Alzheimer’s disease and related conditions. However, these tests are often treated as interchangeable markers of global cognition, despite likely differences in their dependence on specific brain systems, limiting interpretability across studies and clinical contexts. To address this gap, we examined associations between four commonly used cognitive measures—fluid cognition, crystallized cognition, TMT-A, and TMT-B—and multimodal MRI metrics in 725 healthy volunteers aged 36 to 100 years from the Human Connectome Project–Aging. Voxel-wise diffusion MRI and vertex-wise cortical thickness and volume analyses were adjusted for age, sex, and years of education. Higher crystallized and fluid cognition scores and faster TMT-A/B completion times were generally associated with greater white matter integrity. TMT-B showed the most extensive diffusion and cortical associations, involving major projection, commissural, and association pathways and frontoparietal and temporo-occipital cortices. TMT-A and crystallized cognition demonstrated intermediate, overlapping patterns, whereas fluid cognition showed only focal brainstem and limited cortical correlates. These findings demonstrate systematic differences in the neuroanatomical substrates underlying commonly used cognitive tests and provide normative structure–cognition reference maps that can improve test selection, mechanistic interpretation, and sensitivity to brain health in studies of aging, vascular risk, and preclinical neurodegenerative disease.

## 1. Introduction

Cognitive performance in adulthood and aging reflects the integrity of distributed neural systems [1,2], yet different cognitive domains vary markedly in how strongly they depend on the underlying brain structure [3]. This variability has important implications for aging research, where subtle declines in processing speed, executive function, and cognitive flexibility often precede memory impairment and can signal early neurocognitive vulnerability [4]. Identifying which cognitive tests most closely track brain morphology and microstructural integrity is therefore essential for interpreting cognition-MRI relationships in healthy adults and for selecting sensitive measures in studies of aging and preclinical neurodegenerative disease.

The National Institutes of Health (NIH) Toolbox provides harmonized cognitive assessments widely used across large cohort studies and clinical research [5]. Its two primary composite scores are fluid and crystallized cognition. These scores capture distinct cognitive constructs with different developmental and aging trajectories. Fluid cognition reflects novel problem solving, working memory, and processing efficiency, typically peaking in early adulthood before declining with age [6]. In contrast, crystallized cognition reflects accumulated knowledge, vocabulary, and semantic memory, and remains relatively stable or increases across the lifespan [7]. Although these composites are increasingly used in aging and Alzheimer’s disease research [8,9], their precise correlations with underlying structural brain integrity in healthy adult remains poorly defined.

In addition, executive measures such as the Trail Making Test (TMT) A and B offer complementary information that may be more sensitive to morphological and microstructural changes. TMT-A primarily assesses processing speed and visual attention [10], whereas TMT-B additionally requires cognitive flexibility and task-switching [11], functions heavily dependent on long range white matter pathways and frontoparietal cortical networks [12,13]. Importantly, TMT-B performance is a robust predictor of cognitive decline and is frequently used in studies of mild cognitive impairment and Alzheimer’s disease [14,15]. However, despite their widespread use in aging and neurodegenerative research, the normative structural brain characteristics and neuronal pathways supporting NIH Toolbox cognition and TMT performance across healthy adulthood have not been comprehensively characterized.

Because these cognitive measures are widely used in aging and Alzheimer’s disease research [14,15], establishing their normative structural correlates in healthy adults is essential for interpreting deviations observed in clinical and preclinical neurodegenerative cohorts. Yet, prior work has not directly compared the structural brain correlates of fluid cognition, crystallized cognition, TMT-A, and TMT-B using whole-brain voxel-wise diffusion imaging and cortical morphometry within a single large, demographically representative cohort.

In this study, we investigated how four widely used cognitive measures: fluid cognition, crystallized cognition, TMT-A, and TMT-B map onto white matter microstructure and cortical morphology in 725 healthy adults aged 36 to 100 years from the Human Connectome Project Aging. We hypothesized that cognitive tests differ substantially in their dependence on underlying structural integrity, with more complex executive functions requiring broader and more integrated neural networks. By establishing a multimodal structural baseline of cognition across adulthood, our goal was to provide normative benchmarks that can guide the selection and interpretation of cognitive measures in aging research and in studies investigating early neurodegenerative change.

## 2. Materials and Methods

### 2.1. Participants

In our study, we used the Human Connectome Project (HCP) dataset release, previously described by Bookheimer et al. (2019) [16] and accessible through the following link (https://www.humanconnectome.org/ accessed on 27 January 2026). HCP participants were recruited from four representative US sites: Washington University at St. Louis, the University of Minnesota, Massachusetts General Hospital, and the University of California, Los Angeles. The dataset included 725 individuals aged 36–100 years old from the HCP database. The research team excluded any individual with a neurodevelopmental, neuropsychiatric, or neurological disorder history. We focused our analysis on subjects who underwent neuroradiologic examination and neuropsychological tests, including the NIH Toolbox composite scores and the TMT-A and -B. Any subjects who had corrupted metadata, any missing imaging information, or those who failed the imaging processing pipeline were also excluded from our analysis. Demographic variables such as age, sex, and years of education were included as covariates in our models. The analysis included these variables to adjust for potential confounding effects on white matter microstructure and morphological changes. All participants analyzed provided informed consent before participation in this study. In addition, all experimental procedures were approved by the institutional review board.

### 2.2. Neurocognitive Tests

The NIH Toolbox Composite scores assess multiple cognitive domains, including executive function, episodic memory, language, processing speed, working memory, and attention, providing a comprehensive measure of overall cognition across the lifespan [17]. Our analysis utilized the unadjusted fluid cognition (FC) Composite score, with a focus on executive function, and the unadjusted crystallized cognition (CC) Composite score, with an emphasis on language function [18]. We also analyzed the Trail Making Test [19,20], which measures multiple executive functions, such as processing speed and working memory. TMT-A primarily represents processing speed and visual attention [10], whereas TMT-B specifically assesses aspects of cognitive set-shifting [21]. Generally, higher scores on the NIH Toolbox Cognition Battery represent better cognitive performance. On the other hand, higher scores on the TMT-A and -B indicate a longer completion time, reflecting poorer performance [18]. All these scores have demonstrated strong test–retest and interrater reliabilities [22,23].

### 2.3. Neuroimaging Protocol

Structural and diffusion-weighted images of subjects were acquired on a Siemens 3T scanner using a 32-channel head coil (https://www.humanconnectome.org/hcp-protocols-ya-3t-imaging, accessed on 27 January 2026). For structural imaging, T1W acquisitions were achieved with magnetization-prepared rapid gradient-echo (MPRAGE) sequence in sagittal orientation (repetition time = 2400 ms, echo time = 2.14 ms, TI = 1000 ms, flip angle = 8°, field of view = 224 × 224 mm^2^, in-plane matrix = 320 × 320, slice thickness = 0.7 mm, number of slices = 256, voxel size = 0.7 × 0.7 × 0.7 mm^3^). The acquisition time was 7 min and 40 s.

The diffusion-weighted images covering the whole brain were obtained using a spin echobased echo planar imaging sequence. A full diffusion MRI session included 4 runs, including 2 runs for 98 diffusion weighting directions (gradient table: dir98) and 2 runs for 99 diffusion weighting directions (gradient table: dir99), and each run was acquired once using either anterior-to-posterior (AP) or posterior-to-anterior (PA) phase encoding polarity. Each gradient table includes 6 b = 0 acquisitions interspersed throughout each run. Diffusion weighting consisted of 3 shells of b = 1000, 2000, and 3000 s/mm^2^ interspersed with an approximately equal number of acquisitions on each shell within each run (repetition time = 5520 ms, echo time = 89.5 ms, flip angle = 78°, field of view = 210 × 180 mm^2^, in-plane matrix = 168 × 144, slice thickness = 1.25 mm, number of slices = 111, voxel size = 1.25 × 1.25 × 1.25 mm^3^). Each run needs approximately 9 min and 50 s.

### 2.4. Neuroimaging—Diffusion Metrics

All diffusion-MRI (dMRI) scans were preprocessed using the FSL package version 6.0.5 (FMRIB, Oxford, UK). For each subject, diffusion-weighted images with 98 gradient directions were acquired in two phase-encode directions (AP and PA) with opposite polarities. These were then merged to retain SNR benefits and to reduce susceptibility distortions. Non-diffusion-weighted b0 images (b = 0 s/mm^2^) were extracted in a single file and corrected for susceptibility distortions by FSL-topup. FSL-BET [24] was used to remove non-brain tissue and to create a binary mask. Then all the diffusion data was corrected for eddy current distortions using FSL-eddy [25]. This allowed for slice-wise outlier detection and replacement, as well as slice-to-volume registration. FSL-DTIFIT was used to fit a diffusion tensor model at each voxel, reconstructing diffusion tensors and calculating traditional DTI metric maps, including Fractional Anisotropy (FA) and Mean Diffusivity (MD), for subsequent microstructural analysis.

We applied Neurite Orientation Dispersion and Density Imaging (NODDI) multi-compartment models [26], on multi-shell diffusion MRIs to determine the intra-neurite, extra-neurite and free water components. We used accelerated microstructure imaging via a convex optimization tool (dmri-amico 2.0.1) based on Python 3.10.8 with b0_thr = 100 to derive three key NODDI indices of neural tissue at each voxel. The three indices derived were the neurite density (ND) index, reflecting the density of axons or dendrites, the orientation dispersion (OD) index, which establishes the orientation coherence of neurites, and the free water fraction (FWF), which measures the extent of CSF contamination. Excluding those cases of raw data corruption, five DTI indices, FA, MD, ND, OD, and FWF were calculated for a total of 624 subjects.

### 2.5. Neuroimaging—Structural Metrics

All the T1-weighted images were preprocessed in the FreeSurfer v6.0.0 package. Using *recon-all*, for each participant, a two-dimensional cortical surface from a three-dimensional volume was reconstructed in vertex-wise level, and the volume of the cortex was parcellated according to the Destrieux atlas and calculated in Region-of-Interest (ROI)-based level. Besides 74 left and 74 right ROI-based cortical surfaces, the volumes of 45 sub-cortical structures and 70 white matters were collected from the *stats* file for each participant after parcellation and segmentation by FreeSurfer as well.

### 2.6. Tract-Based Spatial Statistics (TBSS)

TBSS was used to improve the sensitivity, objectivity and interpretability of our multi-subject diffusion imaging studies analysis. First, all participants’ FA data was nonlinearly aligned to a standard template space (FMRIB58_FA) to create the mean FA skeleton at a threshold value of >0.2. Afterwards, each participant’s FA data was projected onto the mean FA skeleton, which represented the centers of all tracts common to the group. Similarly, TBSS was also performed for all other diffusion-derived data, including MD, ND, OD and FWF. Finally, a general linear model was built with each cognition index as the independent factor, and with age, gender and years of education as covariates. Voxel-wise statistical analysis of white matter skeleton was conducted with permutation-based nonparametric inference using Randomize (https://fsl.fmrib.ox.ac.uk/fsl/fslwiki/Randomise/UserGuide, accessed on 27 January 2026) to assess the association between 4 cognitive performance tests (including CC, FC, TMTA and TMTB) and our 5 diffusion indices (including FA, MD, ND, OD and FWF). Threshold Free Cluster Enhancement (TFCE) [27] was used to correct for multiple comparisons across the whole brain (*p* < 0.05).

### 2.7. Morphological Measurement Analysis

Linear regression analysis of vertex-wise cortical surfaces for each cognitive performance was performed in FreeSurfer, using age, sex and years of education as covariates. Cluster correction was conducted at a threshold of *p* < 0.05 in both positive and negative directions. Furthermore, we ran a simple linear regression, with age, sex, years of education and total intracranial volume as covariates to observe the correlation between volume ROIs of different brain structures and different cognitive performances. Those ROIs of volume with a *p* < 0.05 after correction by False Discovery Rate (FDR) were retained.

### 2.8. Correlation Analysis Between Morphological Measurement and Diffusion Indices

To explore the associations between fiber pathways microstructure and morphological metrics related to cognitive tests, we determined the correlation between diffusion metrics of white matter tracts and the volumes of different cortical and subcortical gray matter regions, applying FDR correction for multiple comparisons. For DTI metrics, we overlaid 50 tracts from the Johns Hopkins University’s white matter atlas (“JHU-ICBM-DTI-81”) onto each subject’s native diffusion maps in FSL and extracted the mean signal intensity. Morphological measures were obtained using FreeSurfer to calculate the volumes of cortical and subcortical regions. Afterwards, we determined the Pearson’s correlation between these average diffusion metrics from white matter tracts and volumes of cortical and subcortical regions, correcting the *p* values for multiple comparisons by FDR. In addition, we performed multivariable regression to identify the tract-based diffusion metrics and regional volumes independently associated with each cognitive metric, adjusting for age, sex, and years of education.

## 3. Results

### 3.1. Demographic Characteristics

**Figure 1.**
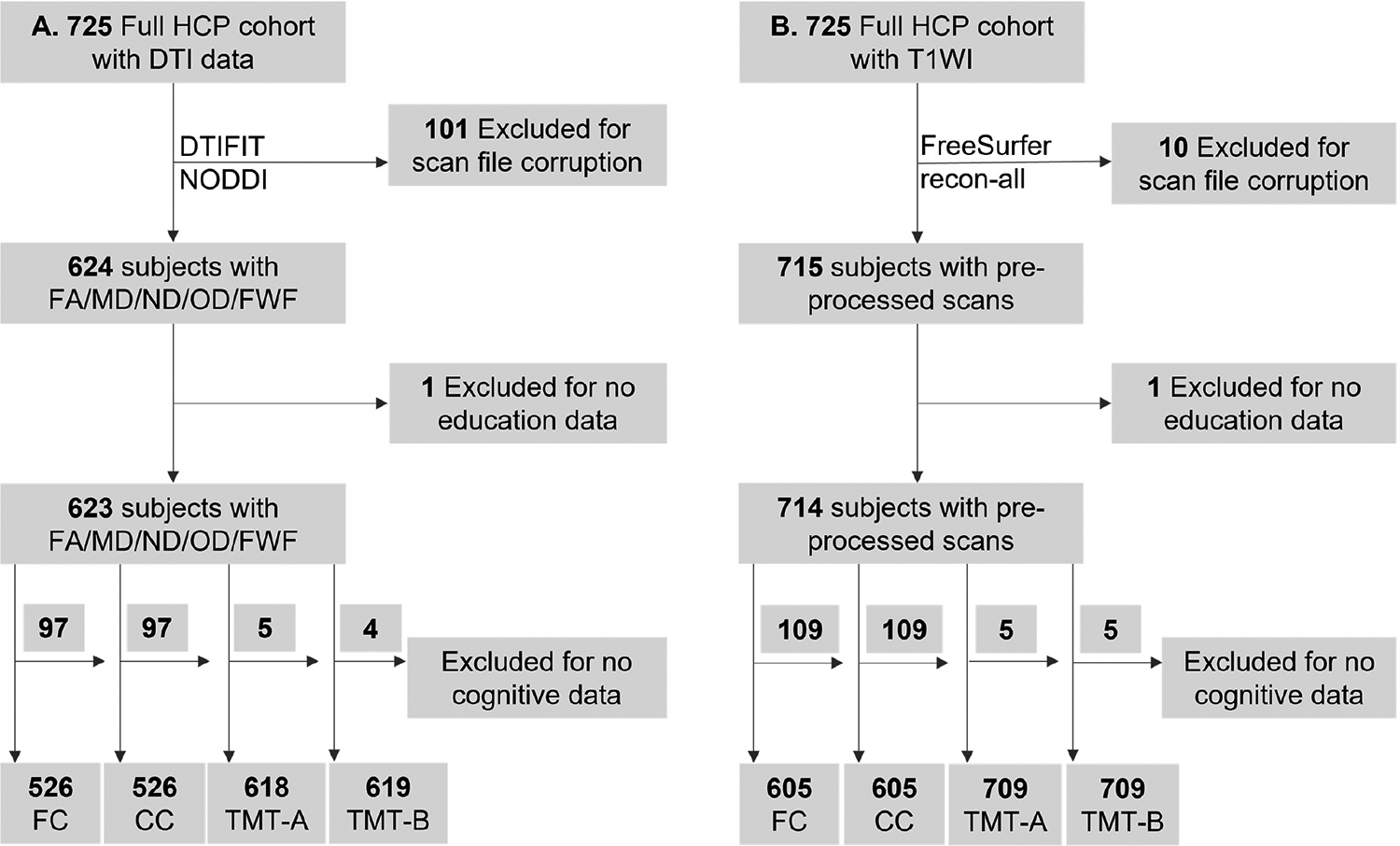
Flowchart of inclusion of subjects with DTI (**A**) or T1-weighted (**B**) images with corresponding cognitive data. CC: crystallized cognition; DTI: diffusion tensor images; FA: Fractional Anisotropy; FC: fluid cognition; FWF: free water fraction; HCP: Human Connectome Project; MD: Mean Diffusivity; ND: neurite density index; NODDI: Neurite Orientation Dispersion and Density Imaging; OD: orientation dispersion index; TMT: Trail Making Test.

Table 1 summarizes the demographic characteristics of all 725 subjects in HCP. From HCP study participants, 526 to 709 subjects were included in our analyses. Figure 1 shows the flowcharts for the inclusion of subjects in the analysis of diffusion and morphological neuroimaging metrics. Each participant in the dataset underwent two diffusion MRI acquisitions (dir_98 with 98 diffusion directions and dir_99 with 99 diffusion directions). We initially processed both sets of scans using DTIFIT and NODDI pipelines. Due to corrupted or incomplete files, diffusion processing was successful in 624 participants for the dir_98 acquisition and in 578 participants for the dir_99 acquisition. To maximize sample size and ensure consistency across analyses, all diffusion MRI analyses were therefore performed using the dir_98 dataset, yielding a final diffusion MRI sample of 624 participants for our analysis. Supplementary Table S1 provides details of demographics in subgroups used in each imaging modality analysis.

### 3.2. White Matter Microstructural Correlates of Cognitive Metrics

**Figure 2.**
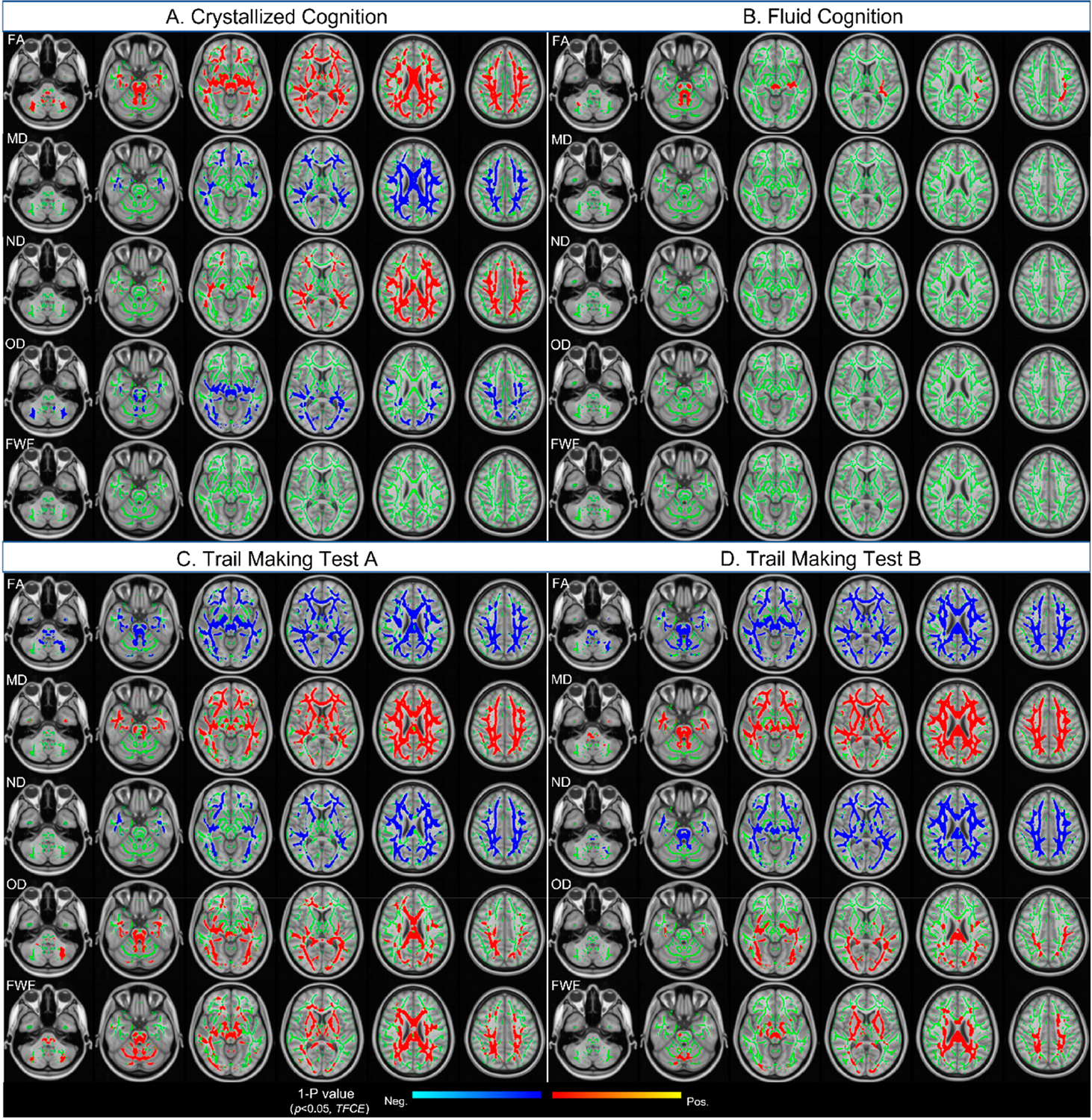
Voxel-wise associations between DTI/NODDI metrics and four cognitive measures (**A**–**D**) assessed using TBSS. Maps display 1-*p* values from voxel-wise regression analyses, corrected for multiple comparisons using threshold-free cluster enhancement (TFCE; *p* < 0.05), with age, sex, and years of education included as covariates. Statistical results are projected onto the mean FA skeleton representing main white matter tracts (shown in green; FA threshold = 0.2). Significant positive and negative associations are shown using red–yellow and light blue–blue color scales, respectively, corresponding to voxels surviving the *p* < 0.05 threshold.

As shown in Figure 2, overall higher scores in crystallized and fluid cognition and lower scores in TMT-A and -B were associated with higher FA and ND but lower MD, FWF and OD, representing greater white matter microstructural integrity. Table 2 lists the top 14 white matter tracts and related diffusion metrics that were significantly associated with the four cognitive tests, after adjusting for age, sex and years of education as covariates.

Crystallized cognition was associated with microstructural integrity of projection tracts in the (anterior, superior, and posterior) corona radiata, posterior thalamic radiation, and middle cerebellar peduncle. There was also an association with microstructural integrity of commissural tracts in the (genu, body, and splenium of) corpus callosum and association tracts in the superior longitudinal fasciculus (Figure 2A, Table 2a).

Fluid cognition was associated with higher FA in projection tracts of the cerebral peduncles, medial lemniscus, and superior cerebellar peduncle, as well as association tracts of the left superior longitudinal fasciculus (Figure 2B, Table 2b).

TMT-A test was associated with the microstructural integrity of projection tracts in the (anterior, superior, and posterior) corona radiata, the posterior thalamic radiation, and the middle cerebellar peduncle. There was also an association with microstructural integrity of commissural tracts in the (genu, body, and splenium of) the corpus callosum, as well as association tracts in the superior longitudinal fasciculus. Compared to crystallized cognition, a higher number of diffusion metrics per white matter tracts showed a significant relationship with the TMT-A test (Figure 2C, Table 2c).

TMT-B test was associated with microstructural integrity of projection tracts in the (anterior, superior, and posterior) corona radiata, (anterior and posterior) limbs of internal capsule, external capsule, and middle cerebellar peduncle. There was also an association with microstructural integrity of commissural tracts in the (genu, body, and splenium of) the corpus callosum as well as association tracts in the superior longitudinal fasciculus (Figure 2D, Table 2d).

### 3.3. Morphological Correlates of Cognitive Metrics

**Figure 3.**
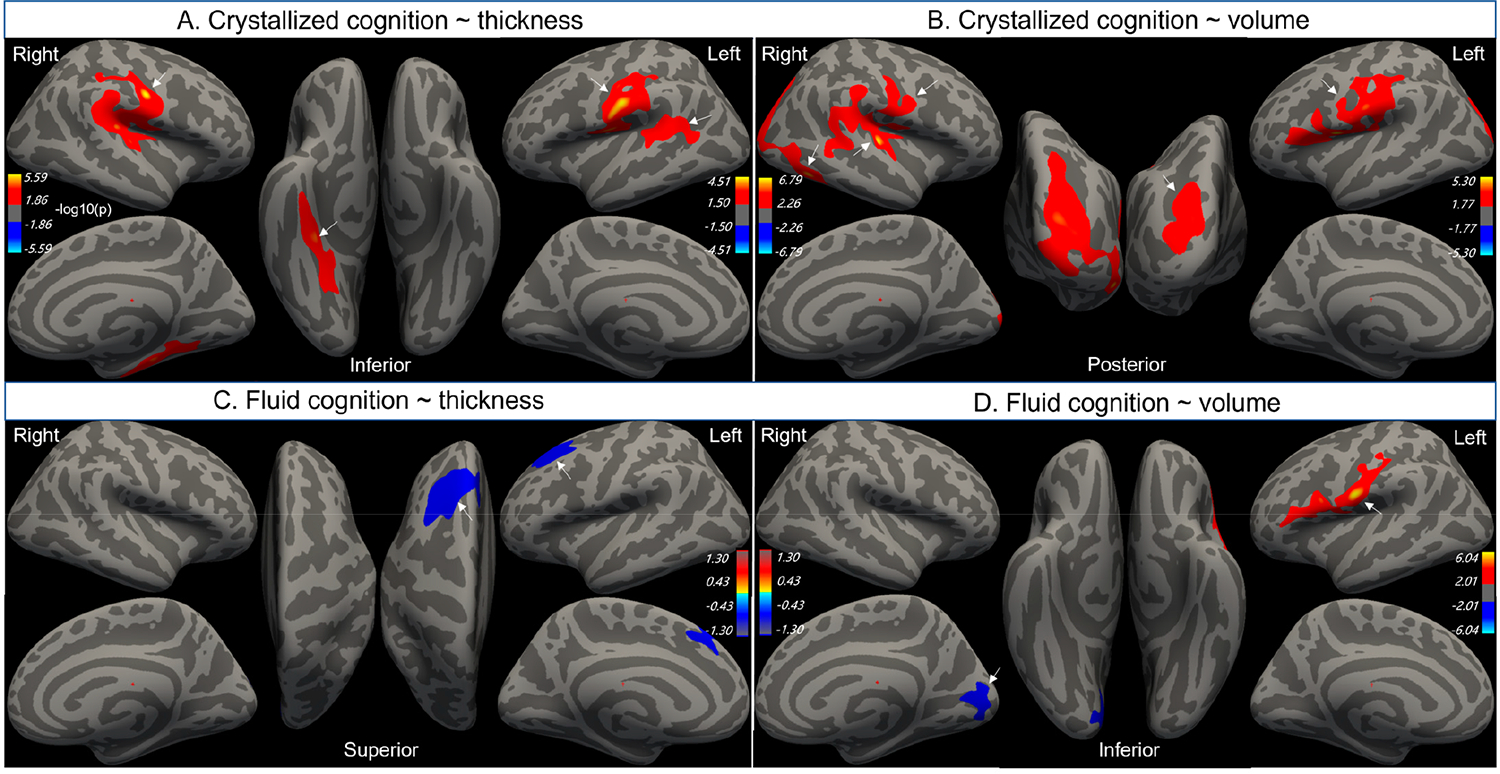
Vertex-wise regression analysis of crystallized (**A**,**B**) and fluid cognition (**C**,**D**) with cortical thickness and volume, controlling for age, sex and years of education (cluster corrected, *p* < 0.05). Arrows indicate cortical regions showing statistically significant associations, which are listed in Table 3. The light blue-to-yellow color scale represents −log10(*p*) values, with warmer colors indicating greater statistical significance.

Figure 3 shows the results of our vertex-wise analysis of cortical thickness and volume in relation to target cognitive tests. The results were adjusted for age, sex and years of education as covariates. Table 3 lists the corresponding cortical gyri, the cluster coordinate and extent found in the vertex-wise analysis.

Crystallized cognition was associated with larger precentral gyri and thicker post-central gyri, inferiorly, in both hemispheres (Figure 3A,B, Table 3a). The inferior pre- and post-central gyri represent the primary motor and somatosensory cortex of the face and tongue, respectively. In addition, crystallized cognition was associated with larger left lateral occipital, right inferior temporal and right transverse temporal gyri, as well as with thicker left supramarginal and right parahippocampal gyri. The transverse temporal (Heschl’s) and supramarginal gyri are implicated in auditory and language processing, respectively. Meanwhile, the lateral occipital and inferior temporal gyri are both involved in visual object recognition tasks.

Fluid cognition was associated with a larger inferior left postcentral gyrus and a smaller right lingual and left superior frontal gyri (Figure 3C,D, Table 3b). The lingual gyrus is implicated in visual function, while the superior frontal gyrus is involved in working memory and executive control [28].

**Figure 4.**
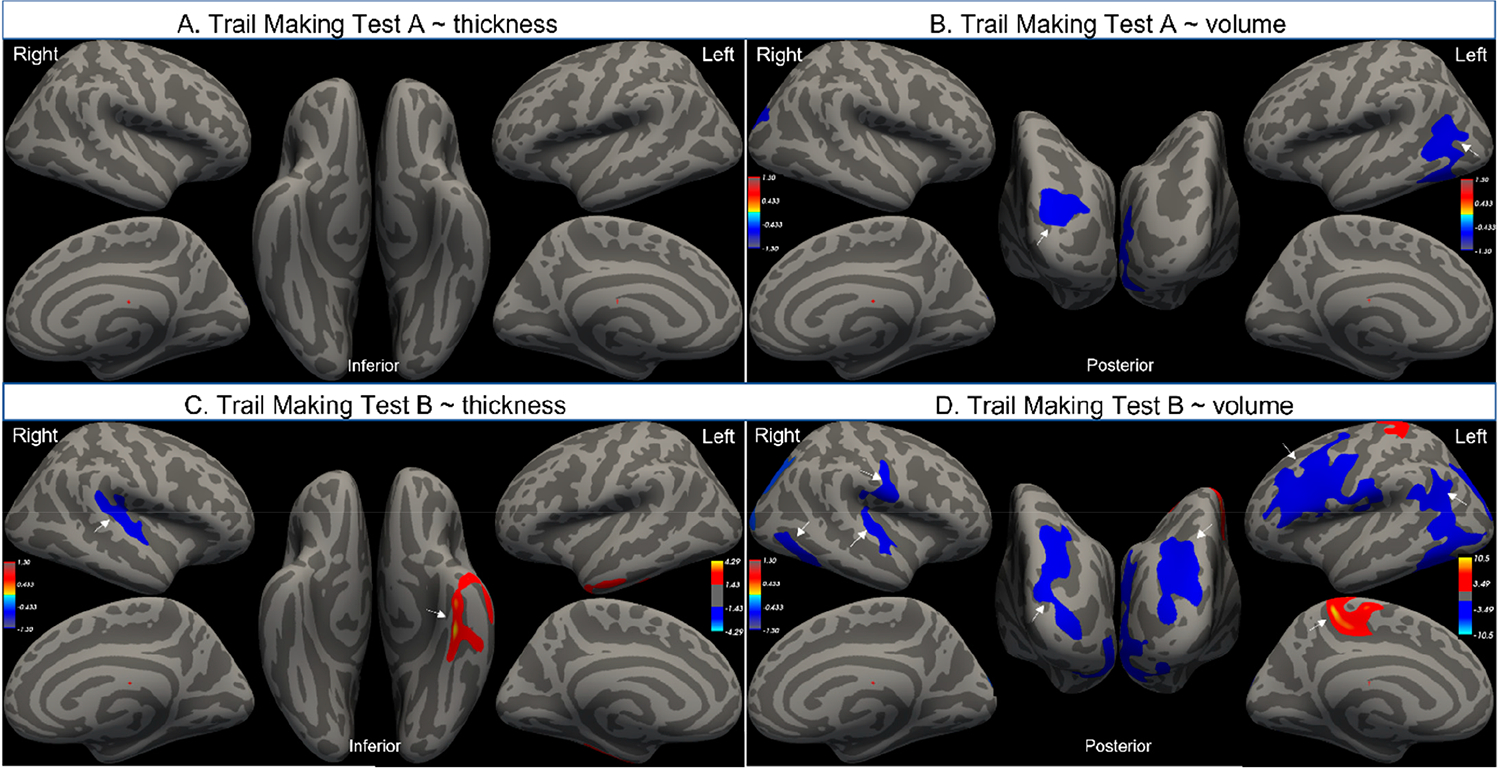
Vertex-wise regression analysis of Trail Making Test A (**A**,**B**) and B (**C**,**D**) with cortical thickness and volume, controlling for age, sex and years of education (cluster corrected, *p* < 0.05). The arrows denote the regions with significance which are listed in Table 3. The values in the light blue–yellow scale denote −log10|*p*|.

Higher TMT-A scores (worse performance) were associated with a smaller left inferior parietal lobule and right lateral occipital gyrus (Figure 4A,B, Table 3c). The inferior parietal lobule is implicated in spatial awareness and attention functions [29].

Higher TMT-B scores (worse performance) were associated with a smaller left precentral, supramarginal and superior parietal gyri, as well as with smaller right postcentral, transverse temporal, inferior temporal and lateral occipital gyri (Figure 4C,D, Table 3d). The superior parietal lobule is implicated in working memory and somatosensory integration [30]. Higher TMT-B scores were also associated with a thinner right superior temporal gyrus, but also associated with a thicker left fusiform gyrus. The superior temporal gyrus is involved in language processing [31], while the fusiform gyrus is implicated in facial recognition tasks.

### 3.4. Relations Between Diffusion Metrics and Morphologic ROIs

**Figure 5.**
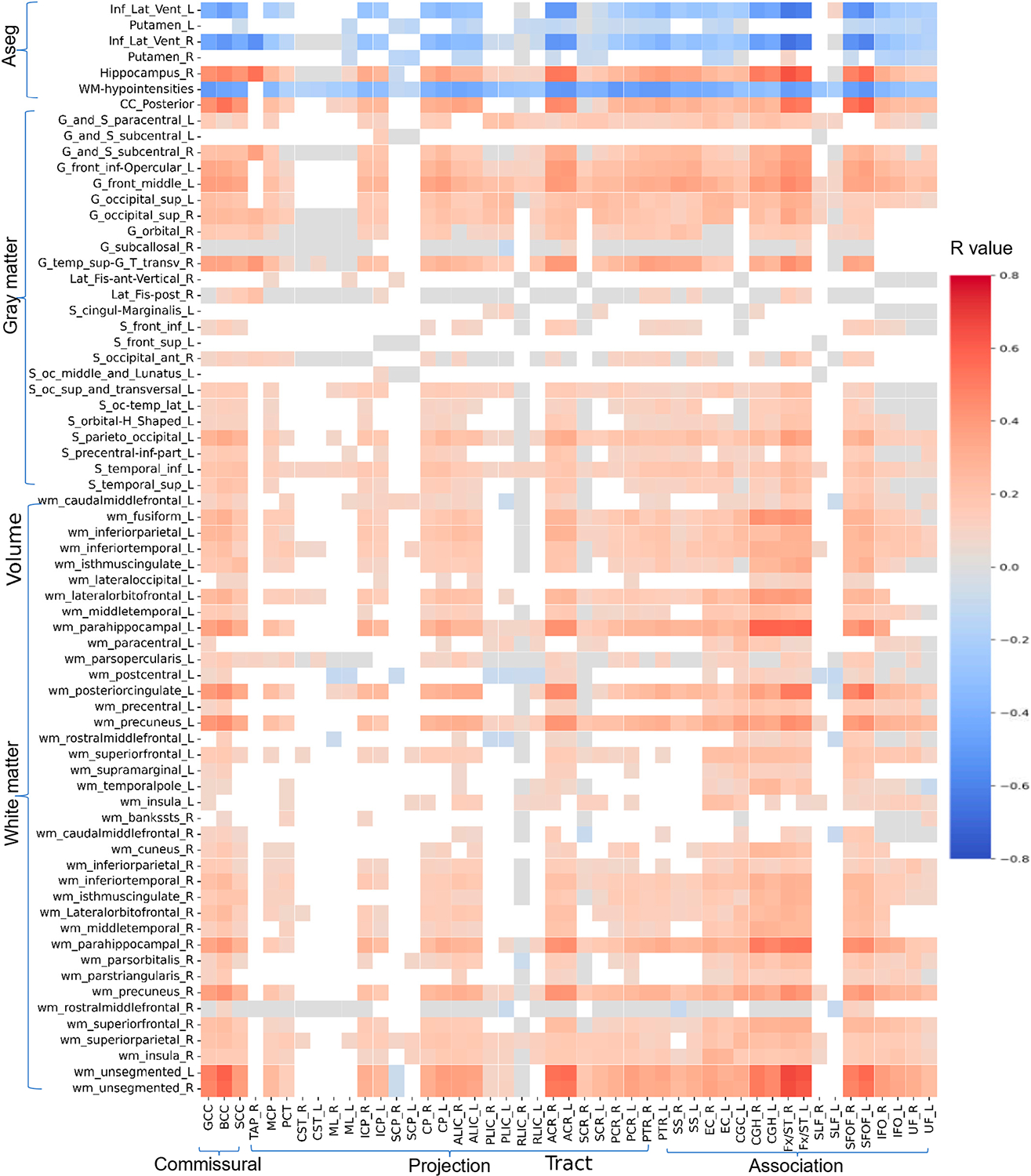
Heatmaps of all correlation coefficient (R) values between volumes of cortical and subcortical regions and white matter tracts FA. Supplemental Figure S1 shows a limited heatmap of the ROIs with absolute r > 0.5 in Pearson’s correlation.

**Figure 6.**
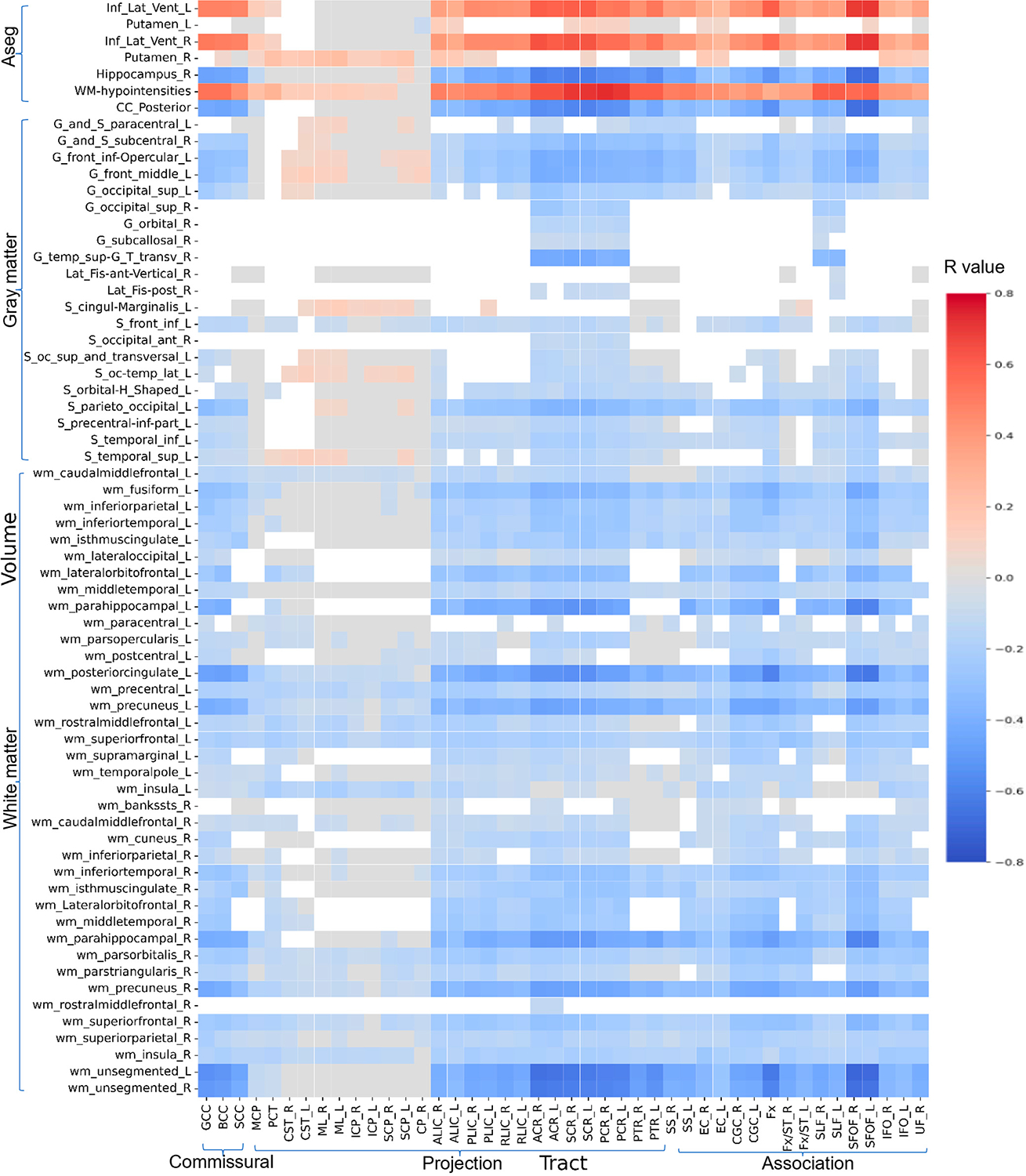
Heatmaps of all correlation coefficient (R) values between volumes of cortical and subcortical regions and white matter tracts MD. Supplemental Figure S1 shows a limited heatmap of the ROIs with absolute r > 0.5 in Pearson’s correlation.

**Figure 7.**
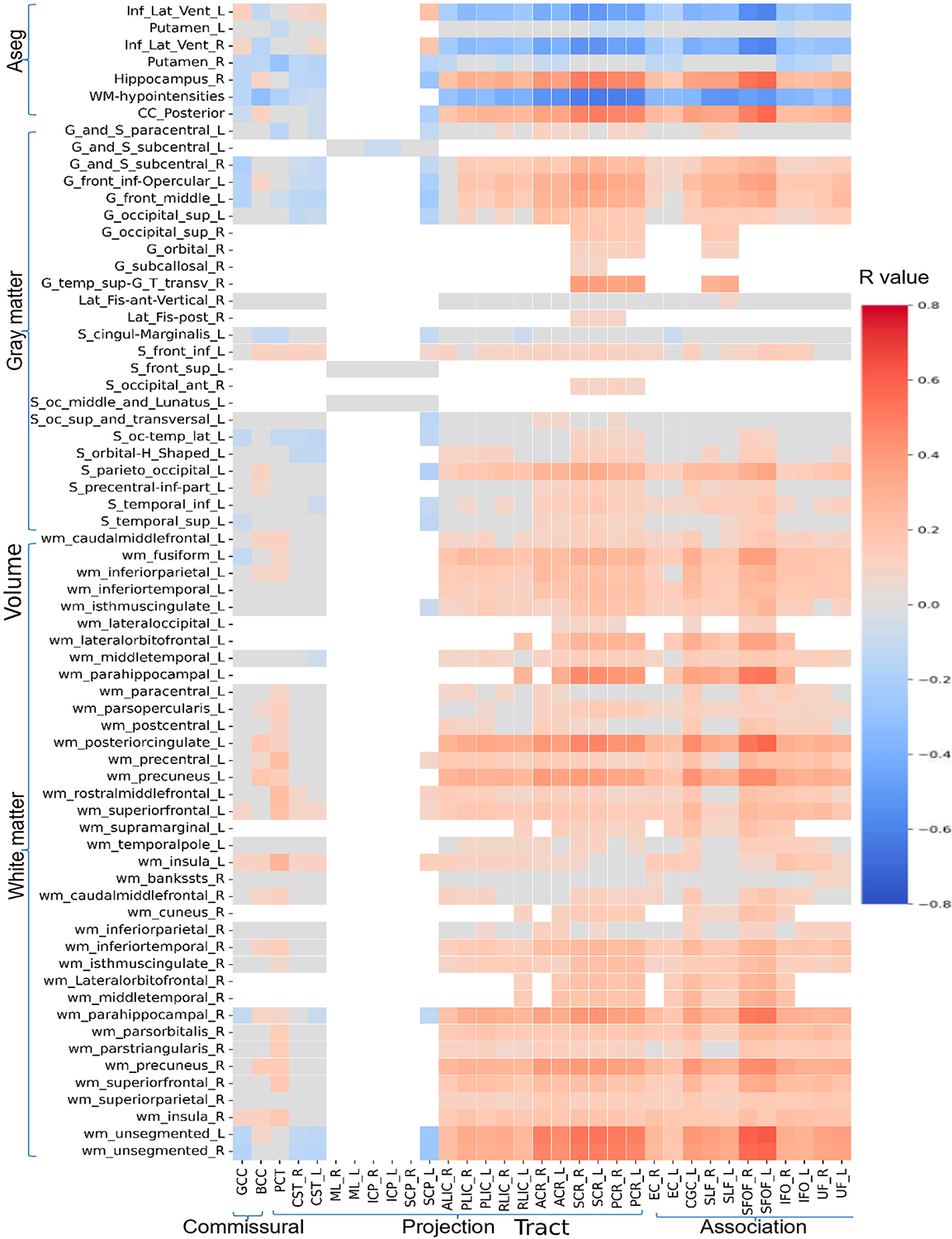
Heatmaps of all correlation coefficient (R) values between volumes of cortical and subcortical regions and white matter tracts ND (*p* < 0.05, FDR corrected). Supplemental Figure S1 shows a limited heatmap of the ROIs with absolute r > 0.5 in Pearson’s correlation.

**Figure 8.**
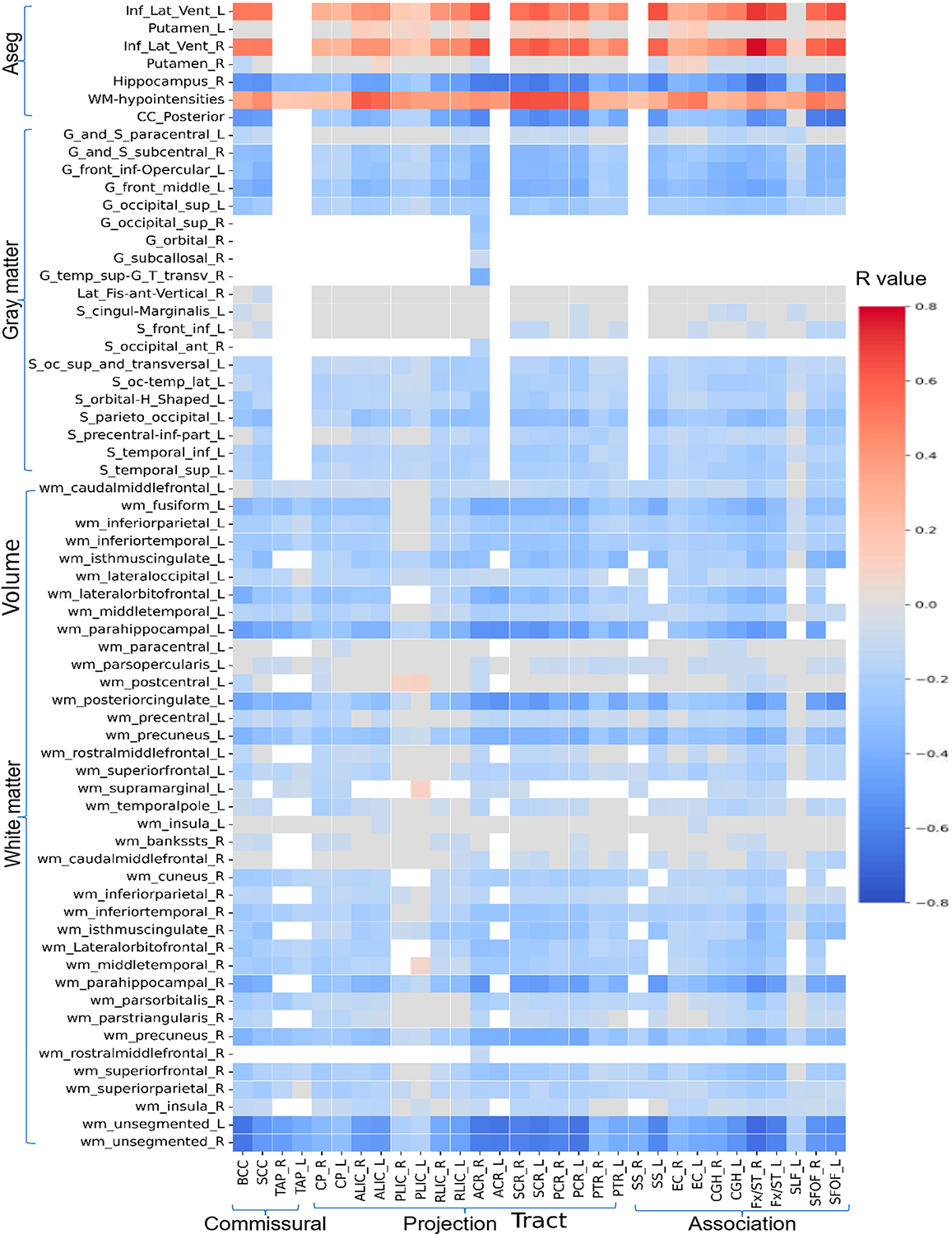
Heatmaps of all correlation coefficient (R) values between volumes of cortical and subcortical regions and white matter tracts FWF (*p* < 0.05, FDR corrected). Supplemental Figure S1 shows a limited heatmap of the ROIs with absolute r > 0.5 in Pearson’s correlation.

Figures 5–8 show heatmaps depicting the relationships between regional brain volumes and different diffusion metrics in the left panel and highlight correlations with significant associations between diffusion and volume metrics and cognitive test performance. Supplementary Table S2 lists all white matter tracts in which mean diffusion metrics showed a significant association with each cognitive performance test after adjusting for age, sex, and years of education as covariates in multivariable regression analyses. Supplementary Table S3a–d lists all cortical and subcortical regions in which regional volumes showed a significant association with each cognitive performance test after adjusting for age, sex, years of education, and total intracranial volume as covariates. Supplementary Table S4a–d reports correlations with significant associations between diffusion and volume metrics and cognitive test performance, with an absolute r > 0.5.

For crystallized cognition, sparse positive correlations were observed between FA and ND values in the body of the corpus callosum, bilateral anterior and superior corona radiata, bilateral superior fronto-occipital fasciculus, and right tapetum, as well as with the volume of the right hippocampus. In contrast, negative correlations were observed with the volumes of the bilateral inferior lateral ventricles and white matter hypointensities. Additional negative correlations were found between the volume of the bilateral inferior lateral ventricles and MD values in the bilateral corona radiata. No significant correlations were observed for fluid cognition or for the OD index.

For both TMT-A and TMT-B, more widespread positive correlations were observed with FA and ND values in the body of the corpus callosum, bilateral corona radiata, fornix/stria terminalis, and superior fronto-occipital fasciculus, as well as with the volumes of the posterior corpus callosum, right hippocampus, and white matter of the bilateral parahippocampal regions and left posterior cingulate. Conversely, negative correlations were observed with the volumes of the bilateral inferior lateral ventricles and white matter hypointensities. These associations were also more pronounced for the MD and free water fraction (FWF) indices. Notably, TMT-A showed stronger correlations with the volume of left parahippocampal white matter, whereas TMT-B showed stronger correlations with the volume of right parahippocampal white matter at this level. In addition, FWF values in the left sagittal stratum and superior fronto-occipital fasciculus were more strongly related to the volumes of specific subcortical regions for TMT-B.

## 4. Discussion

In this large, demographically representative cohort of healthy adults aged 36 to 100 years, we identified distinct patterns of white matter microstructural and cortical morphological associations for four widely used cognitive measures: fluid cognition, crystallized cognition, TMT-A, and TMT-B. Our findings demonstrate that cognitive domains differ markedly in their dependence on structural integrity, revealing a pattern of structure-cognition coupling across healthy adults. Notably, TMT-B exhibited the strongest and most widespread associations with white matter microstructure and cortical morphology, followed by TMT-A and crystallized cognition, whereas fluid cognition showed only focal structural correlates. Together, these results provide a multimodal structural baseline of cognitive performance in normative aging, offering a valuable reference for interpreting MRI-cognition relationships in studies of neurocognitive disorders.

Among all cognitive measures, TMT-B demonstrated the most pervasive associations, involving nearly all major projection, commissural, and association pathways, including the corona radiata, internal capsule, corpus callosum, external capsule, superior longitudinal fasciculus, and cerebellar peduncles. These pathways support cognitive flexibility, tasks-witching, and the integration of information across hemispheres and large-scale cortical systems [32,33]. The strong associations between TMT-B and long-range white matter tracts align with the task’s heavy demands on task shifting and switching. TMT-B also showed extensive cortical associations, including inferior parietal, superior parietal, superior temporal, transverse temporal, inferior temporal, and lateral occipital regions, areas implicated in visuospatial processing, attentional control, language processing, and multisensory integration [34,35]. The breadth of these associations underscores that TMT-B performance reflects coordinated activity across networks, rather than localized brain regions. These findings are consistent with longitudinal aging and neurodegeneration research showing that TMT-B is one of the earliest executive measures to decline with age [36] and is highly sensitive to microstructural disruption in cognitive impairment and related disorders [37]. By mapping TMT-B to its structural substrates in healthy adults, our results establish a crucial normative reference for interpreting deviations in disease populations.

Crystallized cognition exhibited a robust pattern of white matter and cortical associations, though less extensive than those of TMT-B. White matter associations primarily involved the corona radiata, posterior thalamic radiations, corpus callosum, superior longitudinal fasciculus, and middle cerebellar peduncle tracts supporting language, semantic retrieval, and the integration of auditory and visual information [38,39]. Cortical findings further highlighted a strong link to semantic and auditory systems, including thicker cortex in the supramarginal, transverse temporal (Heschl’s), and inferior temporal gyri. These areas play central roles in speech perception, phonological processing, and retrieval of stored knowledge [40–42]. The pattern supports long-standing models of crystallized intelligence as a function of cumulative learning [43]. Given that crystallized cognition remains relatively preserved in healthy aging and often reflects cognitive reserve, these normative structural associations may serve as an anatomical baseline against which to interpret deviations in conditions affecting semantic memory or language networks, including Alzheimer’s disease and progressive aphasias.

In contrast to crystallized cognition and TMT performance, fluid cognition exhibited only focal associations, predominantly within brainstem projection pathways and a limited portion of the left postcentral gyrus. The minimal structural correlates observed here likely reflect several factors, including the fact that fluid cognition declines earlier than crystallized cognition [44]. These results reinforce the notion that fluid cognition may be less suitable as a primary structural MRI correlate in aging research and that functional or multimodal imaging may be required to fully capture its neural correlations.

Our multimodal correlation analyses further clarified how regional volumes relate to tract-specific microstructural integrity. Crystallized cognition showed modest tract–volume relationships, whereas TMT-A and especially TMT-B showed widespread correlations involving the corpus callosum, parahippocampal white matter, posterior cingulate, and hippocampus. These regions are central hubs in memory and executive control networks [45,46] and are among the earliest areas to exhibit microstructural vulnerability in preclinical Alzheimer’s disease [47–49]. We found a positive correlation between the right hippocampus and parahippocampal volume with the FA values of several white tracts, while a negative correlation between bilateral inferior lateral ventral volume and the FA values of these white tracts for CC and TMT-B. These correlations are also likely implicated in aging and Alzheimer’s disease processes [47–49].

Understanding which cognitive tests most closely reflect underlying brain structure is essential for interpreting cognition-MRI relationships in aging studies and for designing clinical trials targeting early neurodegenerative change. Our findings suggest that TMT-B is the most structurally informative cognitive measure and may be the best suited for pairing with diffusion and structural MRI in studies of aging, vascular risk, and preclinical Alzheimer’s disease. Crystallized cognition provides meaningful structural information linked to semantic memory and cognitive reserve. Fluid cognition has limited structural correspondence and may require functional or multimodal imaging for full characterization. By establishing normative structural maps of these cognitive measures, this study provides an anatomical reference framework that can aid researchers and clinicians in distinguishing healthy variation from early pathological change.

The present study has several limitations. First, we used a cross-sectional study design, which limits the ability to assess any aging-related longitudinal changes at an individual-level. Second, only a limited number of potential confounding variables have been recorded in the Human Connectome Project, thus limiting our ability to adjust for all potential confounders. Another limitation in our study is that the tract-based diffusion indices were overlaid on the white matter atlas of “JHU-ICBM-DTI-81”. Because of this, some significant areas in our voxel-wise analysis of white matter tracts are unclassified and not listed in Table 2. Finally, the cognitive tests deduced from the NIH toolbox are continuously evolving, and their measurements may change over time.

## 5. Conclusions

Across a large cohort of healthy adults, we found that cognitive tests vary markedly in how strongly they depend on structural brain integrity. TMT-B showed the most extensive associations with white matter microstructure and cortical morphology, followed by TMT-A and crystallized cognition, whereas fluid cognition demonstrated only focal structural correlates. By establishing normative structure–cognition mappings across healthy adulthood, our findings provide essential benchmarks for interpreting cognitive-MRI relationships in aging research and in studies of preclinical neurodegenerative disease. In particular, the strong structural coupling of TMT-B suggests that it may serve as a sensitive cognitive measure for detecting early disruptions in large-scale neural networks. These results can guide the selection of cognitive tests most aligned with underlying neuroanatomy in both research and clinical settings.

## Supplementary Material

supplementary

**Supplementary Materials:** The following supporting information can be downloaded at: https://www.mdpi.com/article/10.3390/ctn10010005/s1, Table S1. Demographics of the studied population in each sub-dataset; Table S2. Tract (ROI) based diffusion metric correlates with different cognitive tests; Table S3. The cortical and subcortical ROI-based correlates of different cognitive tests; Table S4. Summary of correlation coefficient values (over 0.5) between volumes of cortical and subcortical regions and DTI index of white matter tracts for corresponding cognitive tests (FDR corrected); Figure S1. Heatmap of the ROIs with absolute r > 0.5 in Pearson’s correlation.

## Figures and Tables

**Table 1.** Demographics and characteristics of the studied population.

Characteristic	Total Number of Subjects (n = 725)
Age (years)	60.35 ± 15.73
Education (years)	17.48 ± 2.21
Sex—male	319 (44%)
Race	
White	525 (72.4%)
Black	101 (13.9%)
Asian	52 (7.2%)
more than one race	31 (4.3%)
American Indian	2 (0.3%)
unknown	14 (1.9%)
TMT-A	30.38 ± 12.98
TMT-B	76.52 ± 59.04
Fluid Cognition component	99.11 ± 12.58
Crystalized Cognition component	110.93 ± 9.19

Values are presented as frequency (percentage) or mean ± standard deviation.

**Table 2.** Summary of 1-*p* value maps of top 14 tracts (sorted by minimum *p* values > number of voxels > neuroimaging metrics) from each cluster in regression analysis of DTI indices (TBSS) with crystallized cognition (**a**), fluid cognition (**b**), trail making tests A (**c**) and B (**d**) (corrected by TFCE, *p* < 0.05), adjusting by age, sex and education.

White Matter Tract	Number of Voxels	Minimum *p* Values	Neuroimaging Metrics
**a. Crystallized Cognition**			
Anterior corona radiata—Left	1131;1449;859	0.002;0.01;0.008	FA;MD;ND
Anterior corona radiata—Right	877;1074;583	0.002;0.01;0.008	FA;MD;ND
Body of corpus callosum	1983;2084;842	0.002;0.01;0.008	FA;MD;ND
Genu of corpus callosum	1213;895	0.002;0.01	FA;MD
Middle cerebellar peduncle	1672;871	0.002;0.06	FA;OD
Posterior corona radiata—Left	510	0.008	ND
Posterior corona radiata—Right	543	0.008	ND
Posterior thalamic radiation—Left	639	0.06	OD
Posterior thalamic radiation—Right	614	0.06	OD
Splenium of corpus callosum	1246;1099;1401	0.002;0.01;0.008	FA;MD;ND
Superior corona radiata—Left	969;842	0.01;0.008	MD;ND
Superior corona radiata—Right	1205;1118	0.01;0.008	MD;ND
Superior longitudinal fasciculus—Left	1262;1392;1418;730	0.002;0.01;0.008;0.06	FA;MD;ND;OD
Superior longitudinal fasciculus—Right	1000;1050;1126;539	0.002;0.01;0.008;0.06	FA;MD;ND;OD
**b. Fluid Cognition**			
cerebellar peduncle—Left	105	0.014	FA
cerebellar peduncle—Right	178	0.014	FA
Corticospinal tract—Left	154	0.014	FA
Corticospinal tract—Right	89	0.014	FA
Inferior cerebellar peduncle—Left	89	0.014	FA
Inferior cerebellar peduncle—Right	65	0.014	FA
Middle cerebellar peduncle	146	0.014	FA
Medial lemniscus—Left	130	0.014	FA
Medial lemniscus—Right	105	0.014	FA
Posterior corona radiata—Left	84	0.028	FA
Retrolenticular part of the internal capsule—Left	295	0.042	FA
Superior cerebellar peduncle—Left	195	0.014	FA
Superior cerebellar peduncle—Right	268	0.014	FA
Superior longitudinal fasciculus—Left	574	0.028	FA
**c. Trail Making Test A**			
Anterior corona radiata—Left	1467;1873;1271;802	0.002;0.002;0.004;0.002	FA;MD;ND;FWF
Anterior corona radiata—Right	1180;1423;974;958	0.002;0.002;0.004;0.002	FA;MD;ND;FWF
Body of corpus callosum	2640;2372;402;609;1559;2439	0.002;0.002;0.004;0.004;0.004;0.002	FA;MD;ND;ND;OD;FWF
Genu of corpus callosum	1681;1660;418;696	0.002;0.002;0.004;0.002	FA;MD;ND;FWF
Middle cerebellar peduncle	1853;1186;737	0.002;0.004;0.002	FA;OD;FWF
Posterior corona radiata—Left	500	0.004	ND
Posterior corona radiata—Right	455	0.004	ND
Posterior thalamic radiation—Left	803;771	0.002;0.004	FA;OD
Posterior thalamic radiation—Right	1131;920	0.002;0.004	FA;OD
Splenium of corpus callosum	2033;1517;1841	0.002;0.004;0.002	FA;OD;FWF
Superior corona radiata—Left	1194;1025;737	0.002;0.004;0.002	MD;ND;FWF
Superior corona radiata—Right	1399;1153;769	0.002;0.004;0.002	MD;ND;FWF
Superior longitudinal fasciculus—Left	959;1243;1246;672	0.002;0.002;0.004;0.004	FA;MD;ND;OD
Superior longitudinal fasciculus—Right	1074;1292;1079;356	0.002;0.002;0.004;0.004	FA;MD;ND;OD
**d. Trail Making Test B**			
Anterior corona radiata—Left	1554;1907;1638;173	0.004;0.002;0.004;0.002	FA;MD;ND;FWF
Anterior corona radiata—Right	1456;1490;1238;526	0.004;0.002;0.004;0.002	FA;MD;ND;FWF
Anterior limb of the internal capsule—Right	658	0.002	FWF
Body of corpus callosum	3101;3143;2403;1119;2032	0.004;0.002;0.004;0.008;0.002	FA;MD;ND;OD;FWF
External capsule—Left	576	0.002	FWF
Genu of corpus callosum	1768;1825;978	0.004;0.002;0.004	FA;MD;ND
Middle cerebellar peduncle	2048;1432;1132	0.004;0.002;0.004	FA;MD;ND
Posterior corona radiata—Right	535	0.002	FWF
Posterior limb of the internal capsule—Right	592	0.002	FWF
Splenium of corpus callosum	1916;1948;1369;871;1991	0.004;0.002;0.004;0.008;0.002	FA;MD;ND;OD;FWF
Superior corona radiata—Left	1228;1189;666	0.002;0.004;0.002	MD;ND;FWF
Superior corona radiata—Right	1465;1409;650	0.002;0.004;0.002	MD;ND;FWF
Superior longitudinal fasciculus—Left	1217;1334;1426;649	0.004;0.002;0.004;0.008	FA;MD;ND;OD
Superior longitudinal fasciculus—Right	1184;1400;1344	0.004;0.002;0.004	FA;MD;ND

**Table 3.** Summary of vertex-wise regression analysis of crystallized cognition (**a**), fluid cognition (**b**), trail making tests A (**c**) and B (**d**) with cortical thickness and volume, controlling for age, sex and years of education (cluster corrected, *p* < 0.05).

	Region	Size (mm^2^)	CW-p	CW-pMin	CW-pMax
**a. Crystallized Cognition**					
Thickness	Postcentral—Left	2297.6	0.0002	<0.00001	0.0004
	Supramarginal—Left	1152.77	0.0197	0.01713	0.02227
	Postcentral—Right	3668.81	0.0002	<0.00001	0.0004
	Parahippocampal—Right	1225.96	0.00619	0.00479	0.00759
Volume	Precentral—Left	3898.46	0.0002	<0.00001	0.0004
	Lateral occipital—Left	1164.08	0.0022	0.0014	0.003
	Inferior temporal—Right	3608.25	0.0002	<0.00001	0.0004
	Transverse temporal—Right	2776.65	0.0002	<0.00001	0.0004
	Precentral—Right	935.86	0.01216	0.01017	0.01415
**b. Fluid Cognition**					
Thickness	Superior frontal—Left	1647.91	0.0012	0.0006	0.0018
Volume	Postcentral—Left	2150.13	0.0002	<0.00001	0.0004
	Pericalcarine—Right	775.51	0.04097	0.03744	0.04449
**c. Trail Making Test A**					
Volume	Inferior parietal—Left	1795.43	0.0002	<0.00001	0.0004
	Lateral occipital—Right	841.55	0.02445	0.02168	0.02721
**d. Trail Making Test B**					
Thickness	Fusiform—Left	1194.65	0.01594	0.01375	0.01832
	Superior temporal—Right	1378.1	0.00519	0.004	0.00659
Volume	Precentral—Left	5533.29	0.0002	0	0.0004
	Supramarginal—Left	3693.57	0.0002	0	0.0004
	Paracentral—Left	2128.27	0.0002	0	0.0004
	Superior parietal—Left	1885.82	0.0002	0	0.0004
	Lateral occipital—Right	1632.86	0.0002	0	0.0004
	Inferior temporal—Right	916.82	0.01395	0.01177	0.01613
	Transverse temporal—Right	858.95	0.02247	0.0199	0.02524
	Postcentral—Right	785.5	0.03823	0.03469	0.04175

CW-p: mean cluster-wise *p* value; CW-pMin: minimum cluster-wise *p* value; CW-pMax: maximum cluster-wise *p* value.

## Data Availability

All data used in the present study are available for download from the Human Connectome Project (www.humanconnectome.org accessed on 27 January 2026).

## Abbreviations

The following abbreviations are used in this manuscript:

NIH: The National Institutes of Health
TMT: Trail Making Tests
HCP: the Human Connectome Project
FC: Fluid Cognition
CC: Crystallized Cognition
MPRAGE: magnetization-prepared rapid gradient-echo
AP: anterior-to-posterior
PA: posterior-to-anterior
dMRI: diffusion-MRI
FA: Fractional Anisotropy
MD: Mean Diffusivity
NODDI: Neurite Orientation Dispersion and Density Imaging
ND: neurite density
OD: orientation dispersion
FWF: free water fraction
TBSS: Tract-based spatial statistics
TFCE: Threshold Free Cluster Enhancement
FDR: False Discovery Rate
ROI: Region-of-Interest
CW: cluster-wise
