# Supplementary material for: Neuroimaging Correlates of the NIH Toolbox Cognition and Trail Making Tests: Normative Benchmarks in Healthy Aging
Source: Clin Transl Neurosci. Author manuscript; Available in PMC 2026 Mar 18. (PMC12993754; doi:10.3390/ctn10010005)
Supplement: supplementary [file NIHMS2153120-supplement-supplementary.pdf]

Supplementary Table S1. Demographics of the studied population in each sub-dataset.

	DTI				T1WI			
	FC (N=526)	CC (N=526)	TMT-A (N=618)	TMT-B (N=619)	FC (N=605)	CC (N=605)	TMT-A (N=709)	TMT-B (N=709)
Age (years)	59.46±14.83	59.53±14.94	60.63±15.64	60.62±15.58	59.12±14.97	59.05±14.87	60.30±15.74	60.23±15.62
Education (years)	17.46±2.23	17.45±2.24	17.45±2.24	17.46±2.24	17.44±2.22	17.45±2.22	17.46±2.22	17.46±2.23
Sex - Male	204(38.8%)	194(36.9%)	243(39.3%)	245(39.6%)	252(41.7%)	252(41.7%)	310(43.7%)	312(44.0%)
Race								
white	380(72.2%)	380(72.2%)	454(73.5%)	455(73.5%)	427(70.6%)	427(70.6%)	512(72.2%)	512(72.2%)
black of African American	70(13.3%)	70(13.3%)	79(12.8%)	78(12.6%)	91(15.0%)	91(15.0%)	101(14.2%)	100(14.1%)
Asian	42(8.0%)	42(8.0%)	46(7.4%)	47(7.6%)	48(7.9%)	48(7.9%)	51(7.2%)	52(7.3%)
more than one race	21(4.0%)	21(4.0%)	25(4.0%)	25(4.0%)	24(4.0%)	24(4.0%)	29(4.1%)	29(4.1%)
American Indian	2(0.4%)	2(0.4%)	2(0.3%)	2(0.3%)	2(0.3%)	2(0.3%)	2(0.3%)	2(0.3%)
unknown	11(2.1%)	11(2.1%)	12(1.9%)	12(1.9%)	13(2.1%)	13(2.1%)	14(2.0%)	14(2.0%)
TMT-A	-	-	30.44±13.00	-	-	-	30.35±12.95	-
TMT-B	-	-	-	76.66±62.03	-	-	-	76.40±59.20
NIH Fluid Cog Comp	99.38±12.68	-	-	-	110.88±9.21	-	-	-
NIH Cryst Cog Comp	-	111.19±9.13	-	-	-	99.09±12.59	-	-

[1] Key: CC: Crystallized Cognition; FC: Fluid Cognition; NIH: National Institutes of Health; TMT: Trail Making Test.

Values are presented as frequency (percentage) or mean ± standard deviation

Supplementary Table S2. Tract (ROI) based diffusion metric correlates of different cognitive tests.

a. Crystallized cognition	FA			FWF			MD			ND			OD		
	Tract	Beta	-log(p)	Tract	Beta	-log(p)	Tract	Beta	-log(p)	Tract	Beta	-log(p)	Tract	Beta	-log(p)
	MCP	0.13	3	ACR_R	-0.18	3	ACR_R	-0.13	2	SCR_R	0.13	2	SCP_R	-0.12	2
	PCT	0.07	1				ACR_L	-0.13	2	SCR_L	0.13	2	SCP_L	-0.13	2
	GCC	0.11	2				SCR_R	-0.15	2	PCR_R	0.11	1	CP_R	-0.11	2
	BCC	0.09	2				SCR_L	-0.15	2	PCR_L	0.11	1	CP_L	-0.13	2
	SCC	0.12	3				PCR_R	-0.14	2	SLF_R	0.12	2	PTR_R	-0.12	2
	CST_R	0.13	3				PCR_L	-0.13	2	SLF_L	0.14	2	PTR_L	-0.10	1
	CST_L	0.15	4				SLF_R	-0.12	2				Fx/ST_R	-0.15	3
	ML_R	0.17	6				SLF_L	-0.14	2				Fx/ST_L	-0.13	2
	ML_L	0.18	6												
	ICP_R	0.17	5												
	ICP_L	0.14	3												
	SCP_R	0.22	8												
	SCP_L	0.20	7												
	CP_R	0.21	7												
	CP_L	0.20	6												
	ALIC_R	0.13	3												
	ALIC_L	0.11	2												
	PLIC_R	0.10	2												
	PLIC_L	0.09	2												
	RLIC_L	0.10	2												
	ACR_R	0.11	2												
	ACR_L	0.09	1												
	SCR_L	0.08	2												
	PCR_R	0.14	3												
	PCR_L	0.15	4												
	PTR_R	0.12	2												
	PTR_L	0.11	2												
	SS_R	0.10	2												
	SS_L	0.10	2												
	EC_R	0.10	2												
	EC_L	0.13	3												
	CGH_R	0.13	3												
	CGH_L	0.11	2												
	Fx/ST_R	0.21	5												
	Fx/ST_L	0.13	2												
	SLF_R	0.11	2												
	SLF_L	0.12	3												
	SFOF_R	0.11	2												
	SFOF_L	0.11	2												
	TAP_R	0.09	1												

b. Fluid cognition	FA			FWF			MD			ND			OD		
	Tract	Beta	-log(p)	Tract	Beta	-log(p)	Tract	Beta	-log(p)	Tract	Beta	-log(p)	Tract	Beta	-log(p)
	ICP_L	0.13	2				ICP_L	-0.15	2	ML_R	0.15	2			
	SCP_R	0.20	4				SCP_L	-0.17	2	ML_L	0.13	2			
	SCP_L	0.16	3							ICP_R	0.14	2			
	SLF_R	0.12	1							ICP_L	0.18	3			
										SCP_R	0.19	3			
										SCP_L	0.19	3			

c. TMT-A	FA			FWF			MD			ND			OD		
	Tract	Beta	-log(p)	Tract	Beta	-log(p)	Tract	Beta	-log(p)	Tract	Beta	-log(p)	Tract	Beta	-log(p)
	MCP	-0.19	5	BCC	0.12	2	PCT	0.09	1	RLIC_L	-0.10	1	PCT	0.10	2
	PCT	-0.11	2	SCC	0.21	5	GCC	0.15	2	ACR_L	-0.11	1	SCC	0.18	4
	GCC	-0.15	3	CP_R	0.12	2	BCC	0.14	2	SCR_R	-0.17	2	SCP_R	0.13	3
	BCC	-0.15	3	CP_L	0.12	2	Fx	0.11	2	SCR_L	-0.17	2	SCP_L	0.12	2
	SCC	-0.20	5	ALIC_R	0.15	3	CST_R	0.10	2	PCR_R	-0.12	2	RLIC_R	0.14	3
	CST_R	-0.14	4	ALIC_L	0.13	2	CST_L	0.10	2	PCR_L	-0.14	2	RLIC_L	0.13	3
	CST_L	-0.14	4	PLIC_R	0.10	2	ALIC_R	0.14	3	EC_L	-0.11	1	PTR_R	0.20	5
	ML_R	-0.17	5	PLIC_L	0.09	1	ALIC_L	0.14	3	CGC_L	-0.12	1	PTR_L	0.16	4
	ML_L	-0.19	5	RLIC_R	0.15	3	PLIC_R	0.13	2	SLF_R	-0.14	2	SS_R	0.16	4
	ICP_R	-0.20	6	RLIC_L	0.10	2	PLIC_L	0.13	2	SLF_L	-0.14	2	SS_L	0.13	3
	ICP_L	-0.17	4	ACR_R	0.19	3	RLIC_R	0.13	2	SFOF_R	-0.14	2	CGH_R	0.10	1
	SCP_R	-0.22	7	ACR_L	0.10	1	RLIC_L	0.14	3	SFOF_L	-0.14	2	CGH_L	0.13	2
	SCP_L	-0.21	6	SCR_R	0.22	5	ACR_R	0.21	4	IFO_R	-0.10	1	Fx/ST_R	0.17	4
	CP_R	-0.16	4	SCR_L	0.20	4	ACR_L	0.20	3				Fx/ST_L	0.16	3
	CP_L	-0.16	4	PCR_R	0.25	6	SCR_R	0.24	5						
	ALIC_R	-0.17	4	PCR_L	0.19	4	SCR_L	0.22	5						
	ALIC_L	-0.13	3	PTR_R	0.15	3	PCR_R	0.21	4						
	PLIC_R	-0.10	2	PTR_L	0.11	2	PCR_L	0.21	4						
	PLIC_L	-0.11	2	SS_R	0.12	2	SS_L	0.11	2						
	RLIC_R	-0.14	4	EC_R	0.14	3	EC_R	0.12	2						
	RLIC_L	-0.16	4	EC_L	0.18	4	EC_L	0.14	3						
	ACR_R	-0.14	3	CGH_R	0.12	2	CGC_R	0.11	2						
	ACR_L	-0.12	2	CGH_L	0.12	2	CGC_L	0.14	2						
	SCR_R	-0.08	1	Fx/ST_R	0.13	2	Fx/ST_L	0.09	1						
	SCR_L	-0.11	2	Fx/ST_L	0.13	2	SLF_R	0.15	3						
	PCR_R	-0.13	3	SFOF_R	0.10	2	SLF_L	0.17	3						
	PCR_L	-0.14	3	TAP_R	0.11	2	SFOF_R	0.19	3						
	PTR_R	-0.18	5	TAP_L	0.11	2	SFOF_L	0.17	3						
	PTR_L	-0.16	4				IFO_R	0.12	2						
	SS_R	-0.14	3				IFO_L	0.09	1						
	SS_L	-0.16	4												
	EC_R	-0.16	4												
	EC_L	-0.18	5												

CGC_L	-0.12	2
CGH_R	-0.12	2
CGH_L	-0.13	2
Fx/ST_R	-0.21	5
Fx/ST_L	-0.20	4
SLF_R	-0.12	3
SLF_L	-0.11	2
SFOF_R	-0.18	5
SFOF_L	-0.15	3
IFO_R	-0.11	2

**d. TMT-B**	**FA**			**FWF**			**MD**			**ND**			**OD**		
	Tract	Beta	-log(p)	Tract	Beta	-log(p)	Tract	Beta	-log(p)	Tract	Beta	-log(p)	Tract	Beta	-log(p)
	MCP	-0.22	7	BCC	0.12	2	MCP	0.14	3	PCT	-0.16	3	SCC	0.14	3
	PCT	-0.19	6	SCC	0.32	12	PCT	0.21	6	GCC	-0.11	2	SCP_R	0.14	3
	GCC	-0.19	5	CP_R	0.12	2	GCC	0.20	4	BCC	-0.13	2	SCP_L	0.11	2
	BCC	-0.20	5	CP_L	0.12	2	BCC	0.22	5	CST_R	-0.13	2	RLIC_R	0.13	2
	SCC	-0.24	7	ALIC_R	0.31	11	SCC	0.19	4	CST_L	-0.13	2	RLIC_L	0.10	2
	CST_R	-0.18	5	ALIC_L	0.25	7	Fx	0.09	1	SCP_L	-0.11	2	PTR_R	0.19	5
	CST_L	-0.16	5	PLIC_R	0.25	9	CST_R	0.19	5	ALIC_R	-0.12	2	PTR_L	0.17	4
	ML_R	-0.18	5	PLIC_L	0.20	6	CST_L	0.14	3	PLIC_R	-0.11	2	SS_R	0.17	4
	ML_L	-0.22	7	RLIC_R	0.22	6	ML_R	0.17	4	PLIC_L	-0.11	2	SS_L	0.18	4
	ICP_R	-0.21	6	RLIC_L	0.19	5	ML_L	0.19	5	RLIC_R	-0.14	3	Fx/ST_R	0.15	3
	ICP_L	-0.21	6	ACR_R	0.18	3	ICP_R	0.12	2	RLIC_L	-0.14	3	Fx/ST_L	0.11	1
	SCP_R	-0.25	9	SCR_R	0.32	10	ICP_L	0.12	2	ACR_R	-0.16	3	UF_L	0.10	1
	SCP_L	-0.22	7	SCR_L	0.31	10	SCP_R	0.11	2	ACR_L	-0.16	3			
	CP_R	-0.18	5	PCR_R	0.32	11	SCP_L	0.13	2	SCR_R	-0.25	6			
	CP_L	-0.21	6	PCR_L	0.29	9	CP_R	0.11	2	SCR_L	-0.25	6			
	ALIC_R	-0.26	9	PTR_R	0.19	5	ALIC_R	0.24	7	PCR_R	-0.16	3			
	ALIC_L	-0.17	4	PTR_L	0.15	3	ALIC_L	0.18	5	PCR_L	-0.19	4			
	PLIC_R	-0.14	3	SS_L	0.14	3	PLIC_R	0.24	7	EC_R	-0.16	3			
	PLIC_L	-0.13	3	EC_R	0.26	9	PLIC_L	0.20	5	EC_L	-0.12	2			
	RLIC_R	-0.16	4	EC_L	0.37	14	RLIC_R	0.21	5	CGC_L	-0.13	2			
	RLIC_L	-0.16	4	CGH_R	0.11	2	RLIC_L	0.21	5	SLF_R	-0.17	4			
	ACR_R	-0.20	5	CGH_L	0.11	1	ACR_R	0.31	8	SLF_L	-0.21	5			
	ACR_L	-0.15	3	Fx/ST_R	0.18	3	ACR_L	0.26	6	SFOF_R	-0.12	2			
	SCR_R	-0.13	3	Fx/ST_L	0.12	2	SCR_R	0.36	12	SFOF_L	-0.16	3			
	SCR_L	-0.16	4	SLF_L	0.13	3	SCR_L	0.35	12	IFO_R	-0.15	3			
	PCR_R	-0.17	4	SFOF_R	0.19	5	PCR_R	0.29	9	IFO_L	-0.12	2			
	PCR_L	-0.17	4	SFOF_L	0.15	3	PCR_L	0.29	9	UF_R	-0.10	2			
	PTR_R	-0.18	5				PTR_R	0.12	2	UF_L	-0.09	1			
	PTR_L	-0.18	5				PTR_L	0.12	2						
	SS_R	-0.17	4				SS_R	0.11	2						
	SS_L	-0.19	5				SS_L	0.14	3						
	EC_R	-0.22	6				EC_R	0.21	6						

EC_L	-0.25	9	EC_L	0.23	7
CGC_L	-0.10	2	CGC_R	0.13	2
CGH_R	-0.13	2	CGC_L	0.16	3
CGH_L	-0.12	2	Fx/ST_R	0.17	4
Fx/ST_R	-0.24	6	Fx/ST_L	0.16	3
Fx/ST_L	-0.22	5	SLF_R	0.21	5
SLF_R	-0.14	3	SLF_L	0.26	9
SLF_L	-0.15	4	SFOF_R	0.24	5
SFOF_R	-0.21	6	SFOF_L	0.22	5
SFOF_L	-0.18	4	IFO_R	0.16	4
IFO_R	-0.15	4	IFO_L	0.15	3
IFO_L	-0.12	3	UF_R	0.10	2
UF_R	-0.11	2			
UF_L	-0.12	2			

Five mean DTI indices of tracts overlaid on JHU-ICBM-DTI-81 atlas correlate to four cognitions (a-d) by univariate linear regression analysis, adjusted with age, sex and years of education ($p<0.05$, corrected by False Discovery Rate (FDR)).

Supplementary Table S3. The cortical and subcortical ROI-based correlates of different cognitive tests.

**a. Crystallized cognition**	**Aseg_volume**			**Lh_GrayVol**			**Rh_GrayVol**			**Wmparc_volume**		
	Volume	Beta	-log(p)	Volume	Beta	-log(p)	Volume	Beta	-log(p)	Volume	Beta	-log(p)
	Hippocampus_R	0.15	2				G_and_S_subcentral_R	0.12	2	wm_parsopercularis_L	0.11	2
	Inf_Lat_Vent_R	-0.15	2				G_occipital_sup_R	0.11	2	Wm_rostralmiddlefrontal_R	-0.11	2
							G_orbital_R	0.11	2			
							G_subcallosal_R	-0.09	1			
							G_temp_sup-G_T_transv_R	0.13	2			
							Lat_Fis-post_R	0.13	2			
							S_occipital_ant_R	0.09	1			

**b. Fluid cognition**	**Aseg_volume**			**Lh_GrayVol**			**Rh_GrayVol**			**Wmparc_volume**		
	Volume	Beta	-log(p)	Volume	Beta	-log(p)	Volume	Beta	-log(p)	Volume	Beta	-log(p)
				G_and_S_subcentral_L	0.13	1						
				S_front_sup_L	-0.13	2						
				S_oc_middle_and_Lunatus_L	-0.13	2						

c. TMT-A	Aseg_volume			Lh_GrayVol			Rh_GrayVol			Wmparc_volume		
	Volume	Beta	-log(p)	Volume	Beta	-log(p)	Volume	Beta	-log(p)	Volume	Beta	-log(p)
	Hippocampus_R	-0.17	2							wm_caudalmiddlefrontal_L	-0.13	3
	WM-hypointensities	0.20	5							wm_fusiform_L	-0.12	2
										wm_inferiorparietal_L	-0.10	2
										wm_inferiortemporal_L	-0.12	2
										wm_lateraloccipital_L	-0.09	1
										wm_lateralorbitofrontal_L	-0.11	2
										wm_middletemporal_L	-0.13	2
										wm_parahippocampal_L	-0.11	2
										wm_parsopercularis_L	-0.15	4
										wm_posteriorcingulate_L	-0.12	2
										wm_precentral_L	-0.09	1
										wm_precuneus_L	-0.14	2
										wm_superiorfrontal_L	-0.16	4
										wm_supramarginal_L	-0.08	1
										wm_insula_L	-0.11	2
										wm_cuneus_R	-0.09	2
										wm_inferiortemporal_R	-0.10	2
										wm_Lateralorbitofrontal_R	-0.13	2
										wm_middletemporal_R	-0.11	2

wm_parsorbitalis_R	-0.11	2
wm_precuneus_R	-0.18	4
wm_superiorfrontal_R	-0.12	2
wm_superiorparietal_R	-0.10	2
wm_unsegmented_L	-0.14	2
wm_unsegmented_R	-0.16	2

**d. TMT-B**	**Aseg_volume**			**Lh _GrayVol**			**Rh _GrayVol**			**Wmparc_volume**		
	Volume	Beta	-log(p)	Volume	Beta	-log(p)	Volume	Beta	-log(p)	Volume	Beta	-log(p)
	Inf_Lat_Vent_L	0.13	2	G_and_S_paracentral_L	0.12	2	G_and_S_subcentral_R	-0.12	1	wm_caudalmiddlefrontal_L	-0.22	8
	Putamen_L	0.13	3	G_front_inf-Opercular_L	-0.13	2	Lat_Fis-ant-Vertical_R	-0.14	2	wm_fusiform_L	-0.12	2
	Inf_Lat_Vent_R	0.18	3	G_front_middle_L	-0.18	3				wm_inferiorparietal_L	-0.11	2
	Putamen_R	0.14	3	G_occipital_sup_L	-0.10	1				wm_inferiortemporal_L	-0.15	4
	Hippocampus_R	-0.21	4	S_cingul-Marginalis_L	0.15	3				wm_isthmuscingulate_L	-0.10	2
	WM-hypointensities	0.30	12	S_front_inf_L	-0.15	3				wm_middletemporal_L	-0.14	3
	CC_Posterior	-0.17	4	S_oc_sup_and_transversal_L	-0.14	3				wm_paracentral_L	0.10	2
				S_oc-temp_lat_L	-0.11	2				wm_parsopercularis_L	-0.19	6
				S_orbital-H_Shaped_L	-0.10	1				wm_postcentral_L	0.11	2
				S_parieto_occipital_L	-0.12	2				wm_posteriorcingulate_L	-0.11	2
				S_precentral-inf-part_L	-0.18	4				wm_precentral_L	-0.15	4
				S_temporal_inf_L	-0.14	3				wm_precuneus_L	-0.14	3
				S_temporal_sup_L	-0.12	2				wm_rostralmiddlefrontal_L	-0.11	2
										wm_superiorfrontal_L	-0.19	5
										wm_temporalpole_L	-0.09	2
										wm_insula_L	-0.17	5
										wm_bankssts_R	-0.08	1
										wm_caudalmiddlefrontal_R	-0.10	2
										wm_inferiorparietal_R	-0.11	2
										wm_inferiortemporal_R	-0.14	3
										wm_isthmuscingulate_R	-0.09	1
										wm_parahippocampal_R	-0.11	1
										wm_parsorbitalis_R	-0.14	3
										wm_parstriangularis_R	-0.09	2
										wm_precuneus_R	-0.13	2
										wm_superiorfrontal_R	-0.11	2
										wm_superiorparietal_R	-0.13	3
										wm_insula_R	-0.10	2
										wm_unsegmented_L	-0.14	2
										wm_unsegmented_R	-0.17	2

The cortical and subcortical ROI-based volumes correlate to four different cognitive performances (a-d) by univariate linear regression analysis, adjusted with age, sex, years of education and total intracranial volume ($p<0.05$, FDR corrected).

Supplementary Table S4. Summary of correlation coefficient values (over 0.5) between volumes of cortical and subcortical regions and DTI index of white matter tracts for corresponding cognitive tests (FDR corrected).

**a. FA**	CC_Posterior	Hippocampus_R	wm_parahippocampal_L	wm_parahippocampal_R	wm_posteriorcingulate_L	wm_unsegmented_L	wm_unsegmented_R	Inf_Lat_Vent_L	Inf_Lat_Vent_R	WM-hypointensities
GCC										-0.5<br>TMTA
BCC	0.55<br>TMTB					0.59;0.60<br>TMTB,TMTA	0.57;0.57<br>TMTB,TMTA		-0.51;-0.51<br>CC,TMTB	
ACR_R		0.52;0.53<br>TMTB,TMTA				0.54;0.55<br>TMTB,TMTA	0.52;0.52<br>TMTB,TMTA	-0.5<br>TMTB	-0.5;-0.52<br>CC,TMTB	-0.51<br>TMTA
ACR_L		0.51;0.52<br>TMTB,TMTA				0.55;0.56<br>TMTB,TMTA	0.52;0.53<br>TMTB,TMTA	-0.5<br>TMTB	-0.51<br>TMTB	-0.5;-0.52<br>TMTB,TMTA
PCR_R										-0.51<br>TMTA
PTR_R										-0.5<br>TMTA
PTR_L										-0.5<br>TMTA
CGH_R		0.51;0.51<br>TMTB,TMTA	0.59<br>TMTA	0.52<br>TMTB		0.51;0.52<br>TMTB,TMTA	0.51;0.52<br>TMTB,TMTA			
CGH_L			0.59<br>TMTA							
Fx/ST_R	0.54<br>TMTB	0.61;0.64; 0.64<br>CC,TMTB,TMTA	0.58<br>TMTA	0.53<br>TMTB	0.51;0.5<br>TMTB,TMTA	0.66;0.66<br>TMTB,TMTA	0.65;0.65<br>TMTB,TMTA	-0.64<br>TMTB	-0.65;-0.67<br>CC,TMTB	
Fx/ST_L	0.52<br>TMTB	0.55;0.58; 0.58<br>CC,TMTB,TMTA	0.59<br>TMTA	0.53<br>TMTB	0.51;0.5<br>TMTB,TMTA	0.64;0.64<br>TMTB,TMTA	0.62;0.61<br>TMTB,TMTA	-0.61<br>TMTB	-0.60;-0.62<br>CC,TMTB	
SFOF_R	0.54<br>TMTB					0.5;0.51<br>TMTB,TMTA		-0.51<br>TMTB	-0.51;-0.53<br>CC,TMTB	
SFOF_L	0.6<br>TMTB	0.52;0.56;0.56<br>CC,TMTB,TMTA			0.57;0.57<br>TMTB,TMTA	0.56;0.56<br>TMTB,TMTA	0.54;0.53<br>TMTB,TMTA	-0.56<br>TMTB	-0.57;-0.60<br>CC,TMTB	-0.53<br>TMTA
TAP_R		0.55<br>CC							-0.55<br>CC	

[1] ACR: Anterior corona radiata; BCC: Body of corpus callosum; CGH: Cingulum (hippocampus); Fx/ST: Fornix/Stria terminalis; GCC: Genu of corpus callosum; PCR: Posterior corona radiata; PTR: Posterior thalamic radiation (include optic radiation); SFOF: Superior fronto-occipital fasciculus; SLF: Superior longitudinal fasciculus; TAP: Tapetum

**b. MD**	Inf_Lat_Vent_L	Inf_Lat_Vent_R	WM-hypointensities	CC_Posterior	Hippocampus_R	wm_parahippocampal_L	wm_parahippocampal_R	wm_posteriorcingulate_L	wm_unsegmented_L	wm_unsegmented_R
GCC		0.52<br>TMTB	0.52;0.54<br>TMTB,TMTA						-0.54;-0.55<br>TMTB,TMTA	-0.5;-0.51<br>TMTB,TMTA
BCC		0.5<br>TMTB	0.51;0.54<br>TMTB,TMTA						-0.52;-0.52<br>TMTB,TMTA	
ACR_R	0.61<br>TMTB	0.61;0.64<br>CC,TMTB	0.61;0.63<br>TMTB,TMTA	-0.55<br>TMTB	-0.57;-0.6;-0.61<br>CC,TMTB,TMTA	-0.51<br>TMTA	-0.51<br>TMTB	-0.53;-0.53<br>TMTB,TMTA	-0.66;-0.67<br>TMTB,TMTA	-0.63;-0.63<br>TMTB,TMTA
ACR_L	0.59<br>TMTB	0.58; 0.61<br>CC,TMTB	0.61;0.63<br>TMTB,TMTA	-0.54<br>TMTB	-0.55;-0.58;-0.58<br>CC,TMTB,TMTA			-0.53;-0.53<br>TMTB,TMTA	-0.65;-0.65<br>TMTB,TMTA	-0.62;-0.62<br>TMTB,TMTA
SCR_R	0.61<br>TMTB	0.6;0.63<br>CC,TMTB	0.67;0.7<br>TMTB,TMTA	-0.59<br>TMTB	-0.58;-0.61;-0.61<br>CC,TMTB,TMTA	-0.53<br>TMTA		-0.55;-0.55<br>TMTB,TMTA	-0.63;-0.63<br>TMTB,TMTA	-0.61;-0.6<br>TMTB,TMTA
SCR_L	0.61<br>TMTB	0.61;0.63<br>CC,TMTB	0.68;0.71<br>TMTB,TMTA	-0.6<br>TMTB	-0.58;-0.61;-0.61<br>CC,TMTB,TMTA	-0.52<br>TMTA		-0.54;-0.55<br>TMTB,TMTA	-0.63;-0.63<br>TMTB,TMTA	-0.6;-0.6<br>TMTB,TMTA
PCR_R	0.52<br>TMTB	0.52;0.55<br>CC,TMTB	0.70;0.73<br>TMTB,TMTA	-0.55<br>TMTB	-0.51;-0.55;-0.55<br>CC,TMTB,TMTA				-0.6;-0.6<br>TMTB,TMTA	-0.59;-0.59<br>TMTB,TMTA
PCR_L	0.53	0.52;0.56	0.68;0.71	-0.53	-0.51;-0.54;-0.55				-0.59;-0.6	-0.57;-0.58

	TMTB	CC,TMTB	TMTB,TMTA	TMTB	CC,TMTB,TMTA				TMTB,TMTA	TMTB,TMTA
PTR_R			0.6<br>TMTB							
PTR_L	0.53<br>TMTB	0.54<br>TMTB	0.6<br>TMTB		-0.55<br>TMTB				-0.55<br>TMTB	-0.53<br>TMTB
RLIC_R			0.52;0.53<br>TMTB,TMTA							
RLIC_L			0.51<br>TMTA							
SS_R			0.5<br>TMTB							
SS_L			0.51;0.52<br>TMTB,TMTA							
Fx	0.6<br>TMTB	0.57<br>TMTB		-0.54<br>TMTB	-0.52;-0.52<br>TMTB,TMTA			-0.59;-0.59<br>TMTB,TMTA	-0.63;-0.64<br>TMTB,TMTA	-0.59;-0.59<br>TMTB,TMTA
SFOF_R	0.7<br>TMTB	0.71<br>TMTB	0.53;0.57<br>TMTB,TMTA	-0.67<br>TMTB	-0.68;-0.68<br>TMTB,TMTA	-0.59<br>TMTA	-0.59<br>TMTB	-0.62;-0.62<br>TMTB,TMTA	-0.7;-0.7<br>TMTB,TMTA	-0.67;-0.67<br>TMTB,TMTA
SFOF_L	0.7<br>TMTB	0.71<br>TMTB	0.56;0.59<br>TMTB,TMTA	-0.68<br>TMTB	-0.68;-0.67<br>TMTB,TMTA	-0.59<br>TMTA	-0.58<br>TMTB	-0.64;-0.64<br>TMTB,TMTA	-0.69;-0.69<br>TMTB,TMTA	-0.66;-0.65<br>TMTB,TMTA
SLF_R			0.58;0.61<br>TMTB,TMTA							
SLF_L			0.59;0.61<br>TMTB,TMTA							

[1] ACR: Anterior corona radiata; BCC: Body of corpus callosum; Fx: Fornix; GCC: Genu of corpus callosum; PCR: Posterior corona radiata; PTR: Posterior thalamic radiation (include optic radiation); RLIC: Retrolenticular part of internal capsule; SCR: Superior corona radiata; SFOF: Superior fronto-occipital fasciculus; SLF: Superior longitudinal fasciculus; SS: Sagittal stratum

**c. ND**	CC_Posterior	Hippocampus_R	wm_parahippocampal_L	wm_parahippocampal_R	wm_posteriorcingulate_L	wm_unsegmented_L	wm_unsegmented_R	Inf_Lat_Vent_L	Inf_Lat_Vent_R	WM-hypointensities
ACR_R						0.5<br>TMTB				-0.54<br>TMTB
ACR_L										-0.53;-0.55<br>TMTB,TMTA
SCR_R		0.51;0.51<br>TMTB,TMTA				0.54;0.54<br>TMTB,TMTA	0.51;0.51<br>TMTB,TMTA	-0.51<br>TMTB	-0.51;-0.52<br>CC,TMTB	-0.59;-0.62<br>TMTB,TMTA
SCR_L	0.51<br>TMTB	0.51;0.51<br>TMTB,TMTA				0.53;0.53<br>TMTB,TMTA	0.5;0.5<br>TMTB,TMTA	-0.51<br>TMTB	-0.51;-0.53<br>CC,TMTB	-0.6;-0.63<br>TMTB,TMTA
PCR_R						0.51;0.52<br>TMTB,TMTA				-0.58; 0.61<br>TMTB,TMTA
PCR_L						0.5;0.51<br>TMTB,TMTA				-0.57;-0.6<br>TMTB,TMTA
SFOF_R		0.53;0.54<br>TMTB,TMTA	0.52<br>TMTA	0.51<br>TMTB	0.53;0.53<br>TMTB,TMTA	0.59;0.59<br>TMTB,TMTA	0.56;0.57<br>TMTB,TMTA	-0.56<br>TMTB	-0.57<br>TMTB	
SFOF_L	0.57<br>TMTB	0.58;0.58<br>TMTB,TMTA	0.53<br>TMTA	0.53<br>TMTB	0.58;0.58<br>TMTB,TMTA	0.62;0.62<br>TMTB,TMTA	0.58;0.58<br>TMTB,TMTA	-0.59<br>TMTB	-0.59<br>TMTB	-0.52<br>TMTA
SLF_R										-0.52<br>TMTA
SLF_L										-0.51;-0.53<br>TMTB,TMTA

[1] ACR: Anterior corona radiata; PCR: Posterior corona radiata; SCR: Superior corona radiata; SFOF: Superior fronto-occipital fasciculus; SLF: Superior longitudinal fasciculus

**d. FWF**	Inf_Lat_Vent_L	Inf_Lat_Vent_R	WM-hypointensities	CC_Posterior	Hippocampus_R	wm_parahippocampal_L	wm_parahippocampal_R	wm_posteriorcingulate_L	wm_unsegmented_L	wm_unsegmented_R
BCC	0.52<br>TMTB	0.51<br>TMTB		-0.5<br>TMTB	-0.52;-0.52<br>TMTB,TMTA				-0.65;-0.65<br>TMTB,TMTA	-0.64;-0.65<br>TMTB,TMTA
SCC	0.51<br>TMTB	0.51<br>TMTB			-0.53;-0.54<br>TMTB,TMTA				-0.51;-0.51<br>TMTB,TMTA	-0.5<br>TMTA
ACR_R	0.62<br>TMTB	0.63; 0.62<br>CC,TMTB		-0.57<br>TMTB	-0.6;-0.62;-0.62<br>CC,TMTB,TMTA	-0.53<br>TMTA	-0.52<br>TMTB		-0.63;-0.64<br>TMTB,TMTA	-0.61;-0.62<br>TMTB,TMTA
ACR_L					-0.64<br>TMTA	-0.54<br>TMTA		-0.53<br>TMTA	-0.65<br>TMTA	-0.61<br>TMTA
SCR_R	0.54<br>TMTB	0.57<br>TMTB	0.63;0.65<br>TMTB,TMTA	-0.51<br>TMTB	-0.6;-0.6<br>TMTB,TMTA	-0.51<br>TMTA			-0.61;-0.61<br>TMTB,TMTA	-0.6;-0.61<br>TMTB,TMTA
SCR_L	0.6<br>TMTB	0.61<br>TMTB	0.61;0.64<br>TMTB,TMTA	-0.58<br>TMTB	-0.63;-0.63<br>TMTB,TMTA	-0.53<br>TMTA	-0.5<br>TMTB	-0.51;-0.5<br>TMTB,TMTA	-0.63;-0.63<br>TMTB,TMTA	-0.62;-0.62<br>TMTB,TMTA
PCR_R		0.53<br>TMTB	0.62;0.64<br>TMTB,TMTA	-0.52<br>TMTB	-0.55;-0.55<br>TMTB,TMTA				-0.58;-0.58<br>TMTB,TMTA	-0.6;-0.6<br>TMTB,TMTA
PCR_L	0.57<br>TMTB	0.59<br>TMTB	0.58;0.61<br>TMTB,TMTA	-0.56<br>TMTB	-0.59;-0.6<br>TMTB,TMTA				-0.65;-0.65<br>TMTB,TMTA	-0.64;-0.64<br>TMTB,TMTA
ALIC_R			0.61;0.61<br>TMTB,TMTA							
ALIC_L			0.56;0.58<br>TMTB,TMTA						-0.51;-0.51<br>TMTB,TMTA	-0.5;-0.51<br>TMTB,TMTA
SS_L	0.64<br>TMTB	0.59<br>TMTB		-0.52<br>TMTB	-0.58<br>TMTB		-0.5<br>TMTB		-0.59<br>TMTB	-0.58<br>TMTB
EC_L			0.52;0.5<br>TMTB,TMTA							
CGH_L	0.5<br>TMTB				-0.54;-0.55<br>TMTB,TMTA				-0.53;-0.53<br>TMTB,TMTA	-0.52;-0.53<br>TMTB,TMTA
Fx/ST_R	0.71<br>TMTB	0.78<br>TMTB		-0.56<br>TMTB	-0.71;-0.71<br>TMTB,TMTA	-0.51<br>TMTA	-0.56<br>TMTB	-0.5<br>TMTB	-0.69;-0.69<br>TMTB,TMTA	-0.67;-0.67<br>TMTB,TMTA
Fx/ST_L	0.64<br>TMTB	0.61<br>TMTB		-0.5<br>TMTB	-0.59;-0.59<br>TMTB,TMTA				-0.61;-0.61<br>TMTB,TMTA	-0.6;-0.6<br>TMTB,TMTA
SFOF_R	0.56<br>TMTB	0.58<br>TMTB	0.5;0.52<br>TMTB,TMTA	-0.61<br>TMTB	-0.57;-0.57<br>TMTB,TMTA				-0.54;-0.53<br>TMTB,TMTA	-0.51;-0.5<br>TMTB,TMTA
SFOF_L	0.65<br>TMTB	0.65<br>TMTB		-0.66<br>TMTB	-0.62<br>TMTB			-0.56<br>TMTB	-0.57<br>TMTB	-0.53<br>TMTB

[1] ACR: Anterior corona radiata; ALIC: Anterior limb of internal capsule; BCC: Body of corpus callosum; CGH: Cingulum (hippocampus); EC: External capsule; Fx/ST: Fornix/Stria terminalis; PCR: Posterior corona radiata; SCC: Splenium of corpus callosum; SCR: Superior corona radiata; SFOF: Superior fronto-occipital fasciculus; SS: Sagittal stratum

There is no R value over 0.5 between any volumes and OD index of any tract for any cognition.

Supplementary Figure S1. Heatmap of the ROIs with absolute r>0.5 in Pearson's correlation.

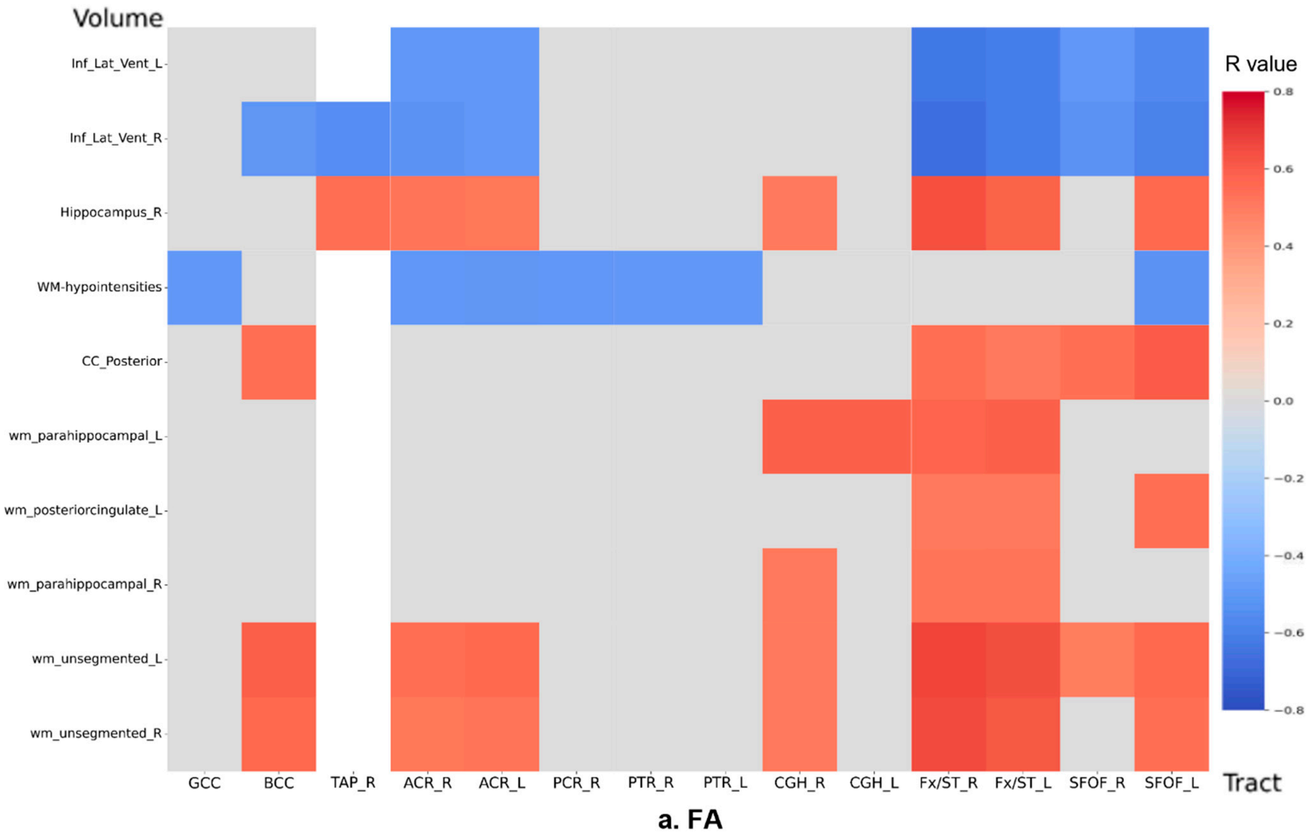

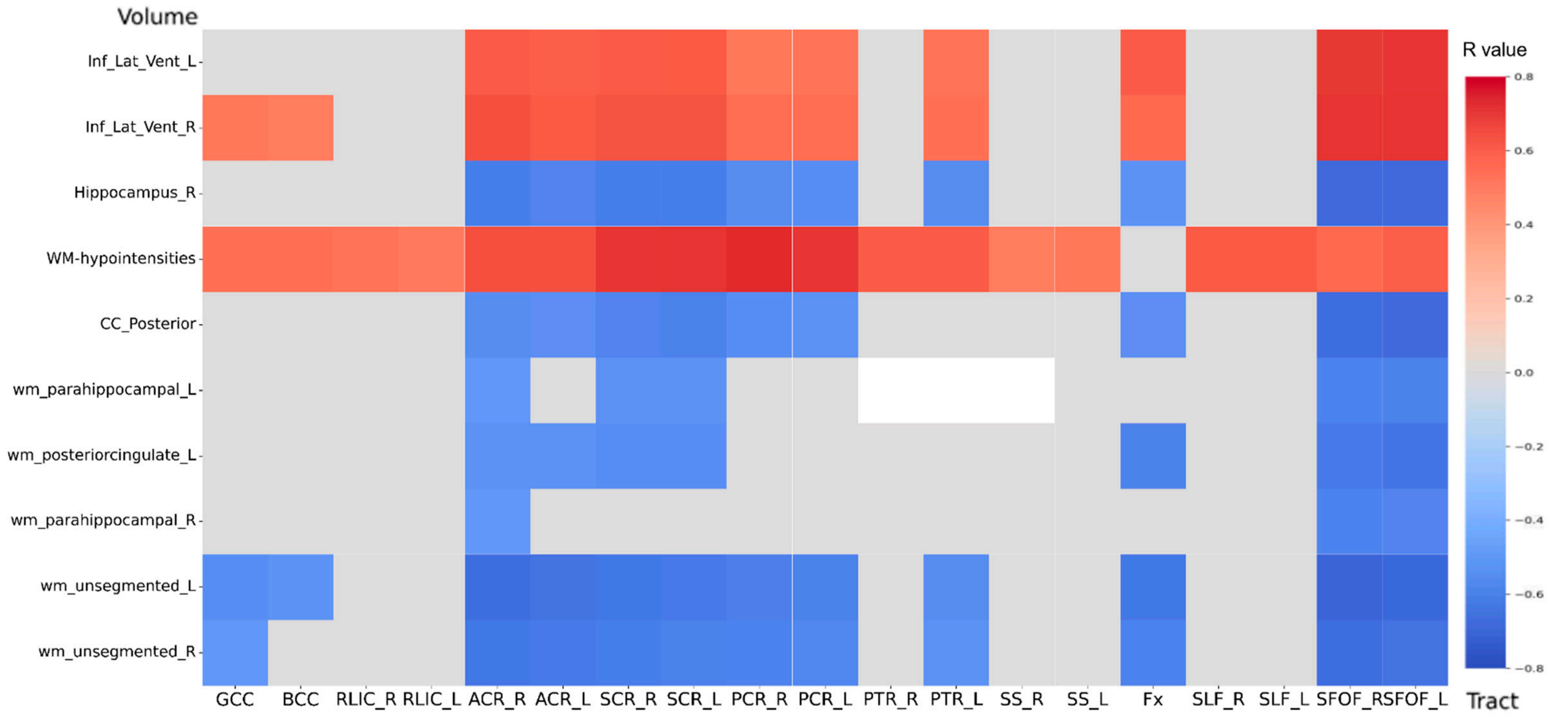
Volume
Inf_Lat_Vent_L
Inf_Lat_Vent_R
Hippocampus_R
WM-hypointensities
CC_Posterior
wm_parahippocampal_L
wm_posteriorcingulate_L
wm_parahippocampal_R
wm_unsegmented_L
wm_unsegmented_R
GCC
BCC
RLIC_R
RLIC_L
ACR_R
ACR_L
SCR_R
SCR_L
PCR_R
PCR_L
PTR_R
PTR_L
SS_R
SS_L
Fx
SLF_R
SLF_L
SFOF_R
SFOF_L
Tract
R value
0.8
0.6
0.4
0.2
0.0
−0.2
−0.4
−0.6
−0.8

b. MD

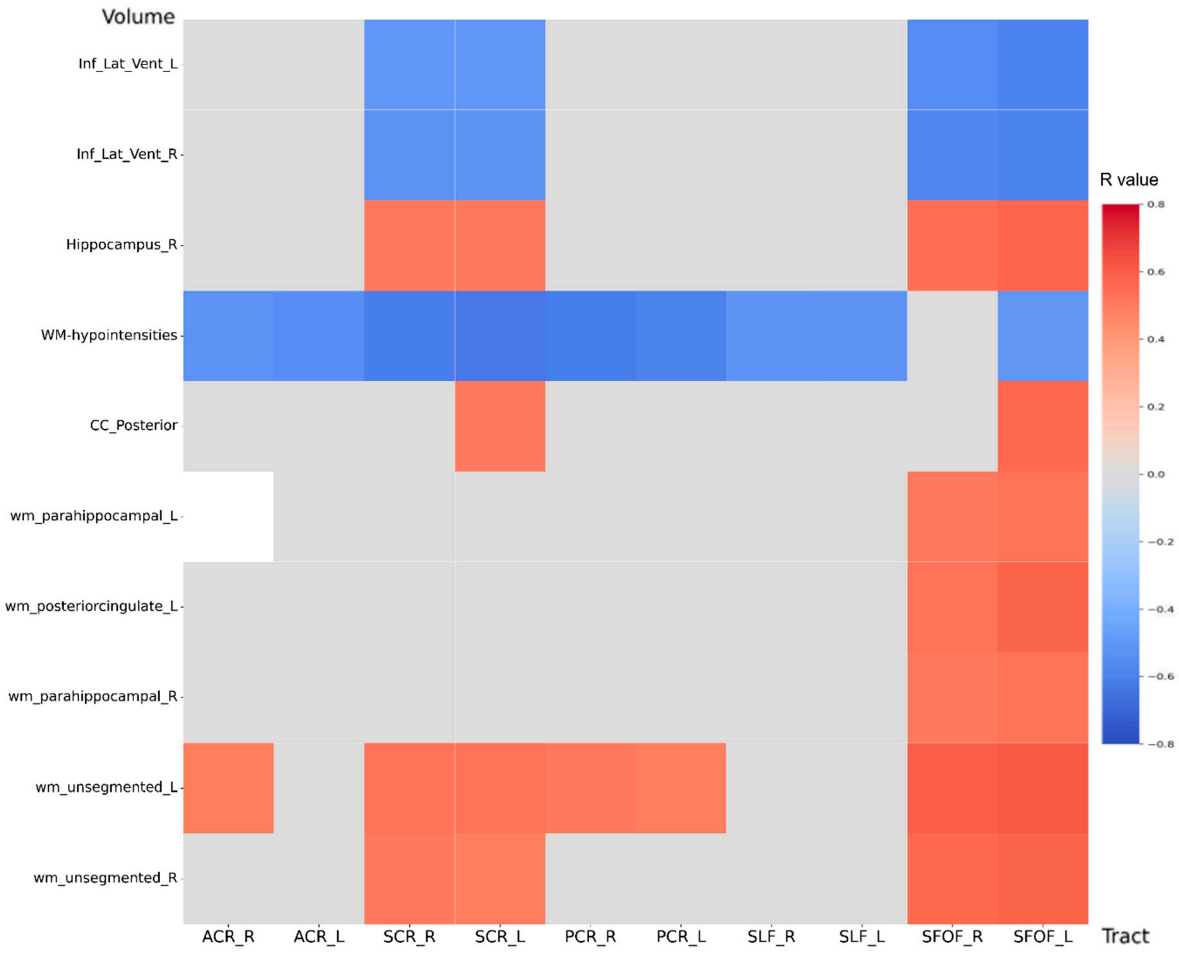
Volume
Inf_Lat_Vent_L
Inf_Lat_Vent_R
Hippocampus_R
WM-hypointensities
CC_Posterior
wm_parahippocampal_L
wm_posteriorcingulate_L
wm_parahippocampal_R
wm_unsegmented_L
wm_unsegmented_R
ACR_R
ACR_L
SCR_R
SCR_L
PCR_R
PCR_L
SLF_R
SLF_L
SFOF_R
SFOF_L
Tract
R value
0.8
0.6
0.4
0.2
0.0
−0.2
−0.4
−0.6
−0.8

c. ND

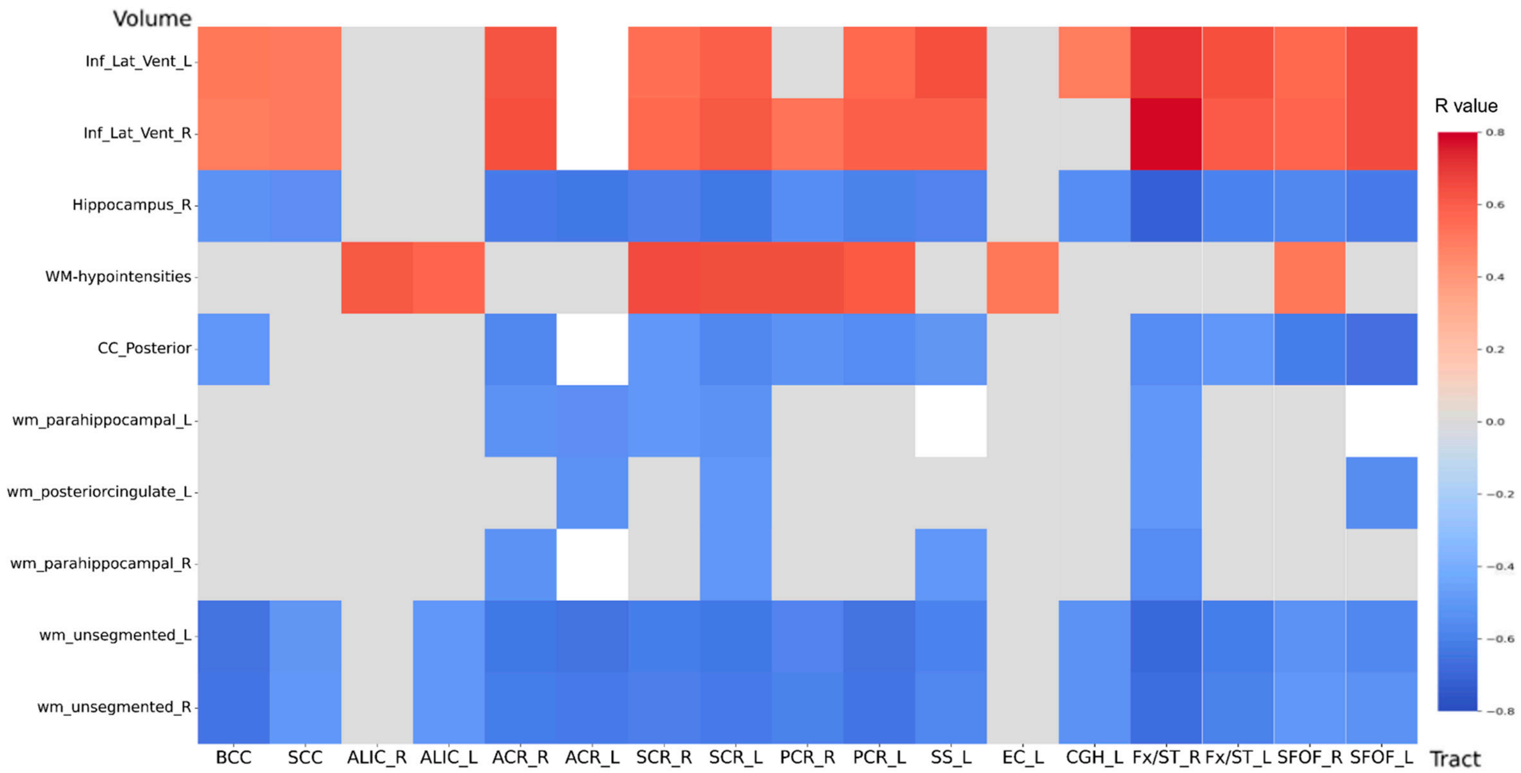
Volume
Inf_Lat_Vent_L
Inf_Lat_Vent_R
Hippocampus_R
WM-hypointensities
CC_Posterior
wm_parahippocampal_L
wm_posteriorcingulate_L
wm_parahippocampal_R
wm_unsegmented_L
wm_unsegmented_R
BCC
SCC
ALIC_R
ALIC_L
ACR_R
ACR_L
SCR_R
SCR_L
PCR_R
PCR_L
SS_L
EC_L
CGH_L
Fx/ST_R
Fx/ST_L
SFOF_R
SFOF_L
Tract
R value
0.8
0.6
0.4
0.2
0.0
−0.2
−0.4
−0.6
−0.8

e. FWF
